# Multiplexed Nanosensors for Real‐Time Monitoring of VOC‐Mediated Signalling Response in Plants

**DOI:** 10.1111/pce.70844

**Published:** 2026-09-02

**Authors:** Mervin Chun‐Yi Ang, Jolly Madathiparambil Saju, Sayyid Mohaideen, Niha Dhar, Sarangapani Sreelatha, Sayuj Koyyappurath, Thomas K. Porter, Gabriel Sánchez‐Velázquez, Vaishnavi Reddy Amarr, Song Wang, Duc Thinh Khong, Suh In Loh, Michael S. Strano, Sarojam Rajani

**Affiliations:** ^1^ Disruptive & Sustainable Technologies for Agricultural Precision IRG, Singapore‐MIT Alliance for Research and Technology Singapore Singapore; ^2^ Natural Sciences and Science Education, National Institute of Education Nanyang Technological University Singapore Singapore; ^3^ Temasek Life Sciences Laboratory Limited 1 Research Link National University of Singapore Singapore Singapore; ^4^ Department of Chemical Engineering Massachusetts Institute of Technology Cambridge Massachusetts USA; ^5^ State Key Laboratory of Mesoscience and Engineering, Institute of Process Engineering Chinese Academy of Sciences Beijing China

## Abstract

To enhance crop performance, intercropping strategies leverage volatile organic compound (VOC)‐driven interactions with companion plants that constitutively emit VOCs. Despite the agricultural importance, the mechanisms and kinetics of VOC‐mediated sensory transduction in receiver plants eavesdropping on neighbouring non‐kin emitters remain largely unknown due to a lack of appropriate non‐destructive analytical tools. In this work, we employ multiplexed salicylic acid (SA) and H_2_O_2_ nanosensors in *Brassica rapa subsp. Chinensis* (pak choy) plants to visualize, in real time, reactive oxygen species (ROS) and SA signal transduction following exposure to constitutively‐released VOCs from neighbouring aromatic plants—namely sweet basil and spearmint. Unique emitter‐specific temporal signatures of ROS and SA were observed in receiver pak choy: sweet basil VOCs induced concomitant generation of ROS and SA at 30 min, whereas spearmint VOCs triggered SA production at 30 min, followed by ROS accumulation. The temporal data enabled the formulation of a diffusion model that quantifies the VOC perception threshold that triggers the distinct early ROS and SA signalling. Transcriptomics analysis at 2 h revealed that both emitters evoke largely distinct changes in pak choy, likely stemming from variations in speed and sequence of the early signal transduction, leading to different phenotypic outcomes. Intercropping with sweet basil led to enhanced pak choy biomass, stress resilience and secondary metabolite accumulation, whereas spearmint as companions had a limited impact. Our study captures in real time, the VOC‐induced rapid signalling in receiver plants and its ensuing effect on growth. These nanosensor‐enabled findings represent an important advance in deciphering how emitter‐specific volatile cues are integrated into plant responses, guiding rational selection of beneficial companion plants for improved yield and nutritional profiles in sustainable agriculture.

## Summary statement

Using multiplexed nanosensors for H_2_O_2_ and salicylic acid, we demonstrate in real‐time the distinct signal transduction induced in Pak choy plants upon perception of constitutively‐emitted VOCs from two different neighbouring companion plants, sweet basil and spearmint, respectively, resulting in different phenotypic outcomes.

## 1 Introduction

Plants emit a diverse array of VOCs, which mediate plant stress responses, their interaction with other organisms and plant‐to‐plant communication (Bouwmeester et al. 2019). More than 1700 plant‐emitted VOCs have been discovered, with each species having a unique VOC profile. Most of these VOCs are constitutively emitted. However, under stress conditions, the emission of VOCs from plants can significantly change both qualitatively and quantitatively (Ninkovic et al. 2019). Plants in natural environments co‐exist in a community and can have different types of neighbours. Studies have demonstrated that both constitutive and stress‐induced VOCs can serve as chemical signals transmitted from emitting plants to receiver plants, mediating interactions between them (Ninkovic et al. 2021). The receiver plants can use these chemical messages to detect the presence of competing neighbours, distinguish between conspecific and heterospecific neighbours (Ninkovic 2003; Vucetic et al. 2014), and as alarm signals for potential pathogen or herbivore dangers (Ninkovic et al. 2019; Loreto and D'Auria 2022). Thus, based on the VOC‐encoded signal, the recipient plants can adjust their growth, defence mechanisms, and overall fitness (Brosset and Blande 2021; Kheam et al. 2024). Studies indicate that the efficacy of VOC‐mediated communication between plants can be genotype‐specific and context‐dependent (Ninkovic et al. 2021; Kheam et al. 2024; Moreira and Abdala‐Roberts 2019; Kong et al. 2024). Research on VOC‐mediated plant‐to‐plant communication has predominantly focused on defense‐related responses of receiver plants—mainly volatiles induced by herbivory in emitter plants and their ability to initiate defense response in neighbouring undamaged receiver plants (Sakurai and Ishizaki 2024; Kessler et al. 2023; Frank et al. 2021).

Chemical communication among plants plays a significant role not only in natural plant communities but also in agricultural systems. Intercropping or companion planting is a practice gaining attention to achieve effective integrated pest management and increased yields in a sustainable manner (Hall et al. 2023; Toker et al. 2024). Aromatic plants have been used as companion plants to repel insects and herbivores as they constitutively release high amounts of VOCs, which can have considerable implications for the neighbouring plants. For instance, intercropping with aromatic plants in apple orchards decreased herbivore abundance (Beizhou et al. 2012). Volatiles from mint varieties were shown to induce defense response in nearby soybean plants and Komatsuna (*Brassica rapa* var. perviridis) plants reducing herbivore damage (Sukegawa et al. 2018). Similarly, intercropping of aromatic plants in tea plantations facilitated pest suppression (Gong et al. 2024). A recent study further showed that mixed planting with basil plants improved wound response in tomato (Yoshida et al. 2024). These studies demonstrate that volatile signalling plays a significant role in determining the impact of companion planting on the target plant. Even though VOC‐driven plant‐to‐plant interaction is now known, VOC perception, and the mechanisms and kinetics of VOC‐mediated signal transduction in receiver plants, remains elusive. One key reason is that very few receptors of VOCs have been identified, despite plants emitting a large variety of over 1700 VOCs, with at least 38 known to be involved in plant‐to‐plant communication (Brosset and Blande 2021). This suggests that plants might have additional mechanisms for detecting VOCs besides receptor‐mediated, including physical structures such as cuticle, stomata, plasma membrane‐localized transporters or ion channels, and intracellular proteins (Loreto and D'Auria 2022; Arimura and Uemura 2025; Sugimoto et al. 2023; Gong et al. 2023).

Upon VOC perception, a change in the plasma membrane potential of cells resulting in ion flux imbalances, mostly comprising calcium ions (Ca^2+^) is proposed to be among the earliest VOC‐induced responses in plants (Zebelo and Maffei 2016; Zebelo et al. 2012; Asai et al. 2009). Recently, in Arabidopsis, a green fluorescent protein‐based Ca^2+^ biosensor was used to monitor in real time, the spatial and temporal changes in Ca^2+^ signalling following exposure to green leaf volatiles (GLVs) emitted by damaged plants (Aratani et al. 2023). Ca^2+^ is an important secondary messenger involved in multiple stress signalling pathways and is associated with the production of a ROS molecule, H_2_O_2_, which is another key early secondary messenger signalling molecule (Petrov and Van Breusegem 2012). VOCs are also known to mediate redox changes in plants. For instance, exposure to a GLV, *Z*‐3‐hexenyl acetate was reported to induce production of H_2_O_2_ in wheat (Ameye et al. 2020). Exposure to methanol VOC was also shown to induce accumulation of ROS in tomato (Hann et al. 2014) and pinene in Arabidopsis (Riedlmeier et al. 2017). A recent study showed that tomato plants, pre‐exposed to basil essential oils for 15 h, accumulated higher amount of ROS upon wounding than control plants (Yoshida et al. 2024). In these studies, ROS production was typically detected using either 3,3‐diaminobenzidine (DAB) or nitroblue tetrazolium (NBT) chemical staining, which are both destructive and non‐specific methods (Daudi et al. 2012; Martin et al. 2022) and hence not amenable for real‐time monitoring of ROS signalling dynamics in living plants.

Phytohormones play a crucial role in coordinating different signal transduction pathways in plants that control growth and stress response. An increasing number of studies on plant‐derived and microbial VOCs have established that VOC exposure can modulate the phytohormone network in the receiver plants (Brosset and Blande 2021; Erb 2018). For example, GLVs emitted by damaged plants have been shown to promote jasmonic acid (JA) biosynthesis in receiver plants, thereby inducing defense responses. Exposure to monoterpene volatiles, α‐ and β‐pinene, in Arabidopsis triggered SA signalling in Arabidopsis leading to activation of systemic acquired resistance (SAR)‐associated genes (Riedlmeier et al. 2017). Exposure of maize seedlings to VOCs from damaged weeds increased SA content, which led to improved growth and herbivore resistance (Sakurai et al. 2023). Apart from JA and SA, VOCs can also modulate auxin and abscisic acid (ABA) hormonal pathways in plants (Brosset and Blande 2021). Overall, VOCs can presumably trigger different signalling cascades in plants, and crosstalk between these signalling molecules is proposed to help the plant integrate different volatile cues to yield specific responses to a VOC blend (Erb 2018). Deciphering the links between volatile perception and downstream signalling is therefore important to understand how plants translate volatile cues into unique responses in both natural ecosystems and agricultural settings, to improve their resilience and fitness.

To facilitate such advances, non‐destructive and species‐agnostic technologies are required. In recent work, nanobionic plants with surface‐enhanced Raman scattering (SERS) nanoparticles were developed that can detect VOCs. The SERS‐based sensor plants showed that plants are able to capture VOCs emitted from neighbouring plants (Choi et al. 2025). For example, a nanobionic sensor plant placed in proximity to an injured tobacco plant, displayed nicotine‐specific SERS signal on its leaf. However, the downstream signalling upon VOC perception of receiver plants was not studied. Therefore, in this study, we used a nanosensor multiplexing approach (Ang et al. 2024) to analyse in real‐time the dynamics of VOC‐driven downstream sensory transduction in *Brassica rapa subsp. Chinensis* (pak choy) plants, an important leafy vegetable crop, when exposed to constitutively‐emitted VOCs from two different aromatic plants, namely spearmint and sweet basil (Figure 1a).

**Figure 1:**
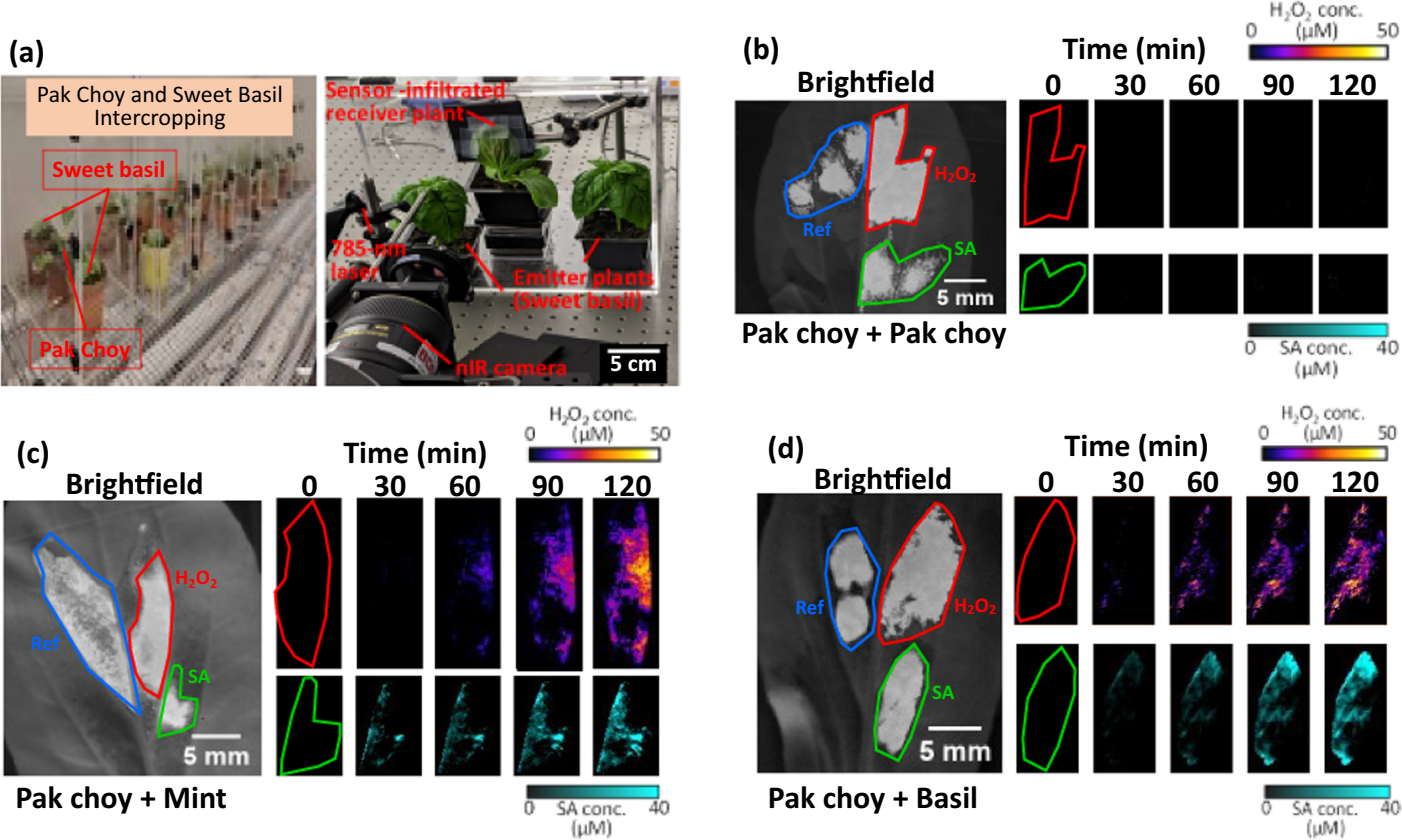
(a) Photographs showing pak choy intercropping with sweet basil plants in custom‐made transparent acrylic chambers (left) and sensor‐infiltrated pak choy intercropping with sweet basil plants in transparent acrylic chamber, under 785 nm laser excitation of sensor near‐infrared fluorescence (right). Scale bar: 5 cm; Representative bright‐field images and concentration maps of H_2_O_2_ (top row) and SA (bottom row) for pak choy plants with different companion plants, namely (b) 2 pak choy, (c) 2 spearmint, and (d) 2 sweet basil.

As generation of H_2_O_2_ and SA has been observed as a VOC‐induced response in receiver plants, we used single‐walled carbon nanotube (SWNT) based optical nanosensors that have been developed for real‐time detection of H_2_O_2_ and SA (Ang et al. 2024; Lew et al. 2020) in planta to probe the spatiotemporal dynamics of their production in response to VOC. Receiver pak choy plants infiltrated with both the nanosensors were placed near spearmint or sweet basil emitter plants for simultaneous monitoring of *in planta* H_2_O_2_ and SA generation upon exposure to the constitutively emitted VOCs from the aromatic plants. We observed for the first time the distinct temporal activation of ROS and SA production in a receiver plant within 2 h, in response to unique blend of VOCs emitted by different neighbouring plants. In contrast, no generation of ROS and SA was observed in receiver pak choy when two pak choy plants were used as neighbouring plants. Further, distinct transcriptomic changes were also found in pak choy leaves in response to VOC exposure from both aromatic plants within 2 h. Pak choy plants growing in the vicinity of sweet basil plants demonstrated an increase in both shoot and root biomass, as compared to pak choy growing beside pak choy or spearmint plants. Additionally, these pak choy plants intercropped with sweet basil showed enhanced accumulation of secondary metabolites and improved resistance to water and nitrogen stress. This work bridges the gap between VOC perception and the resulting internal plant biochemical response. Overall, our study demonstrates that nanosensor multiplexing can be a transformative strategy to investigate the VOC‐induced inter‐plant signal transduction in real time and relate it to the subsequent impact on the receiver plant.

## 2 Results

### 2.1 Detection of Distinct Generation of ROS and SA in Receiver Pak Choy With Different Emitter Companion Plant Using Multiplexed Nanosensors

To probe the role of H_2_O_2_ and SA in sensory transduction of VOCs in plants in real‐time, the SA and H_2_O_2_ sensors were infiltrated into a 3‐week‐old receiver pak choy leaf on the right side of its midvein, partitioned by a dominant leaf vein. Both SA and H_2_O_2_ sensors exhibit selective near‐infrared (NIR) fluorescence quenching in response to respective changes in SA and H_2_O_2_ levels within the plant, while remaining relatively inert to other phytohormones (Ang et al. 2024). The magnitude of fluorescence quenching within the imaged leaf areas can be converted to quantify the local SA and H_2_O_2_ concentrations, using respective sensor calibration curves (Ang et al. 2024). On the left side of the midvein, a reference sensor, which remains invariant to changes in SA and H_2_O_2_ levels, was infiltrated to derive a ratiometric sensor response. Thereafter, the sensor‐infiltrated plant was placed inside a well‐aerated 30 × 30 × 30 cm Perspex box. At *t* = 15 min, different neighbouring plants were introduced and nanosensor fluorescence intensity changes in the receiver pak choy plant were monitored for 2 h. When 2 pak choy plants as neighbouring emitters were placed inside the box on either side of the sensor‐infiltrated receiver pak choy plant, no significant fluorescence changes were observed for both the SA and ROS sensors within the measurement period. By applying the respective sensor calibration curves, the sensor fluorescence maps were converted to local SA and H_2_O_2_ concentration maps (Figure 1b), which illustrates the absence of any changes in both SA and H_2_O_2_ concentration levels within the imaged areas of the leaf. Similar experiments were performed with aromatic plants, spearmint and sweet basil separately, as companion emitter plants instead of pak choy. In contrast to the pak choy plants, with aromatic plants as neighbours, significant fluorescence quenching of both the ROS and SA nanosensors were observed in the pak choy receiver plant, indicative of H_2_O_2_ and SA accumulation (Supplementary Movies 1 and 2). Derived H_2_O_2_ and SA concentration maps obtained from the nanosensors are shown at 30‐min time intervals, along with the brightfield images of corresponding pak choy leaf, upon exposure to spearmint (Figure 1c) and sweet basil (Figure 1d) plant VOCs, respectively.

By averaging across the respective leaf areas infiltrated with H_2_O_2_ and SA sensors, we obtained the time plots of *in planta* relative changes in H_2_O_2_ and SA concentrations, normalized to the basal in planta concentrations at *t* = 0. Figure 2a shows that there are negligible changes in the concentration of both molecules with pak choy as companion plants. While both spearmint and sweet basil companion plants could evoke the production of ROS and SA in the receiver pak choy plant, the temporal kinetics of their generation varied. Additionally, rather than a typical stress‐induced rapid ROS burst (Ang et al. 2024; Lew et al. 2020), VOC‐induced ROS accumulation was observed to be a gradual increase in endogenous H_2_O_2_ levels. When two spearmint plants were introduced as companion emitter plants, the sensors reported a relatively delayed H_2_O_2_ production compared to SA (Figure 2b). When two sweet basil plants were introduced as companion emitter plants, H_2_O_2_ and SA accumulation were both observed simultaneously around 30 min (Figure 2c). With spearmint companion plants, the onset of SA production in receiver pak choy plants was observed at 30 min and H_2_O_2_ generation was observed later at *t* = 53 min (Figure 2d).

**Figure 2:**
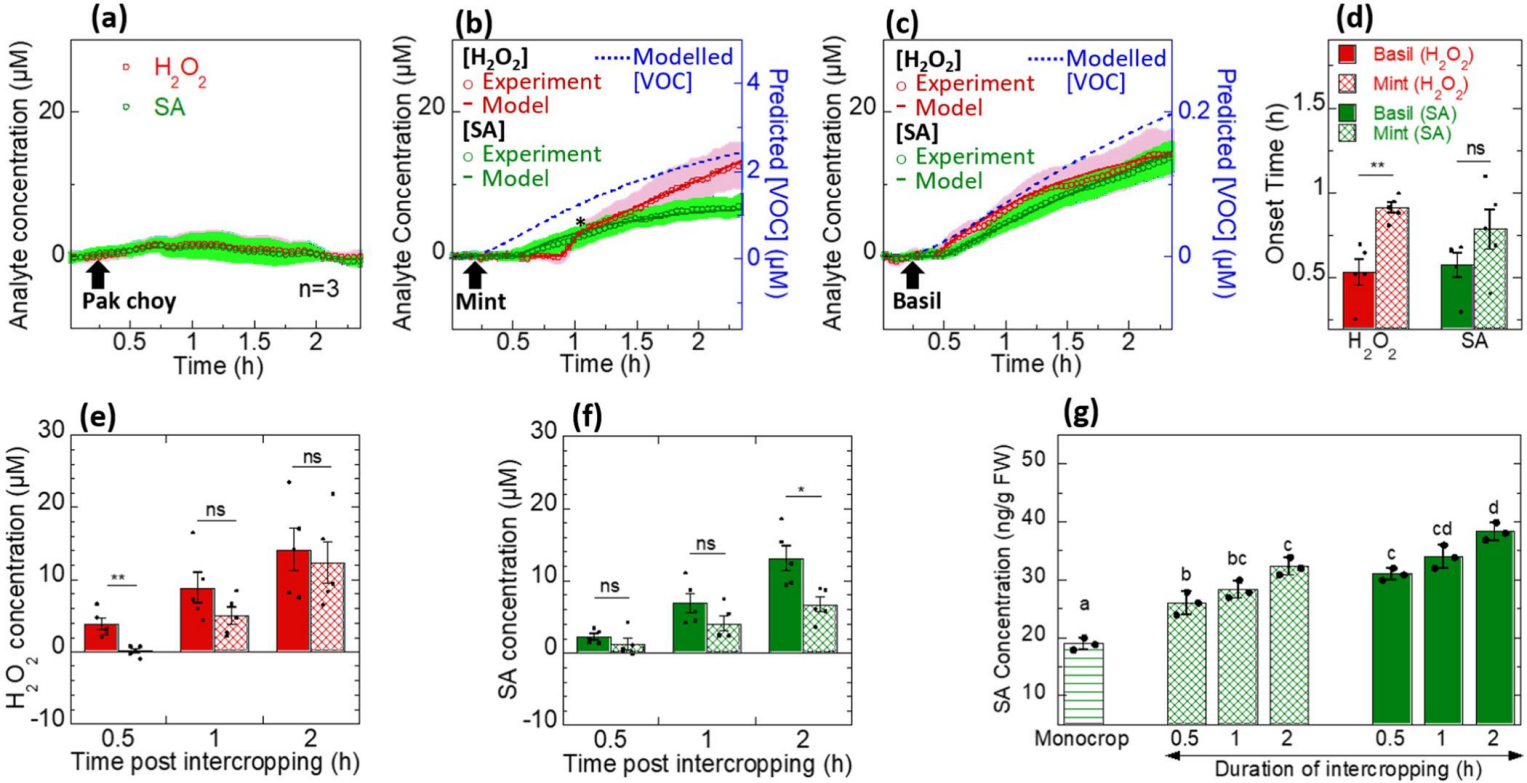
Time‐plots of change in H_2_O_2_ (red) and SA (green) concentration when (a) two pak choy, (b) two spearmint and (c) two sweet basil are introduced at *t* = 15 min (black arrow) as companion plants. Concentrations are derived from either nanosensor experiments (circles) or mathematical modelling (lines). Shaded regions represent standard error across *n* = 3 independent biological replicates for (a) and *n* = 5 independent biological replicates for (b, c). Blue dotted line represents the predicted VOC concentration accumulated at the receiver plant obtained via mathematical modelling. Asterisk (*) represents the timepoint at which there is a change in rate of H_2_O_2_ generation due to negative feedback from SA accumulation; Temporal characterization of (d) Onset of H_2_O_2_ (red) and SA (green) production detected by respective sensors, upon intercropping with sweet basil (block) and spearmint (diagonal stripes), (e) H_2_O_2_ concentration change (red) and (f) SA concentration change (green), in response to sweet basil (block) and spearmint (diagonal stripes) intercropping at 0.5, 1, and 2 h time‐points. Bar graphs show the mean values with error bars representing standard error from independent biological replicates (*n* = 5). Dots represent each data point. Two‐tailed unpaired *t* test, *p < 0.05, **p < 0.01. (g) Average SA concentration in pak choy as determined by LCMS, at *t* = 0.5, 1 and 2 h of exposure to either spearmint (green diagonal stripes) or sweet basil (green block) companion plants. Pak choy plants with pak choy companions are used as monocropped controls (green horizontal stripes). Bar graphs show the mean values with error bars representing standard deviations from independent biological replicates (*n* = 3). Individual data points are shown as dots. Different alphabet letters show significant differences using one‐way ANOVA with a Tukey's HSD test at *p* < 0.05. [Color figure can be viewed at wileyonlinelibrary.com]

Although H_2_O_2_ accumulation in receiver pak choy plants began later than SA when paired with spearmint companion plants, the sensors show that the amount of H_2_O_2_ generated is more than SA by *t* = 2 h. In contrast, with sweet basil companion plants, across the 2 h measurement period, the concentration of ROS and SA generated were similar. At *t* = 2 h, the amount of ROS produced in the receiver pak choy by either aromatic companion plants were similar, reaching 14.1 ± 2.9 μM with sweet basil and 12.4 ± 2.8 μM with spearmint, respectively (Figure 2e). Both companion plants induced SA generation in receiver pak choy at 30 min, but by 2 h, the amount of SA produced is significantly higher with sweet basil as the emitter at 13.1 ± 1.7 μM, compared to spearmint at 6.6 ± 1.0 μM (Figure 2f). We further confirmed the SA sensor results by LC‐MS analysis that there is increased generation of SA in receiver pak choy when in proximity to either spearmint or sweet basil. LC‐MS analysis of Pak choy leaves also shows a higher concentration of SA at 2 h when sweet basil plants were used as companion plants, compared to when spearmint plants were used, further corroborating the trends observed by the SA nanosensor. Hence, we demonstrate, for the first time, the real‐time temporal monitoring of ROS and SA generation in receiver pak choy plants, as part of the VOC‐mediated plant‐to‐plant communication.

The temporal insights from these experiments enabled the formulation of a mathematical model that captures how VOCs travel from an emitter spearmint/sweet basil plant, accumulate at a receiver plant, and initiate downstream H_2_O_2_ and SA signalling in the receiver pak choy plant. We have combined a diffusion model that predicts the VOC accumulation concentration at the receiver plant (Supporting Information, Supplementary Figure 1), and a kinetic model that describes the VOC signal transduction upon recognition in the receiver plant resulting in variable delay of H_2_O_2_ and SA signalling.

The diffusion model assumes that rate of VOC accumulation inside the plant is driven by a concentration gradient between the external VOC levels at the receiver surface and the internal VOC concentration. Once the VOC concentration in the internal compartment of the receiver plant exceeds a threshold value, downstream signalling, such as ROS or SA production, is triggered. The time taken for reaching this threshold concentration value is hence a key determining factor for the onset of ROS or SA production. We have also logically deduced from the model that VOC emission and perception is temperature‐ and distance‐dependent. The diffusion model predicts that at ambient temperature (298.15 K) and inter‐plant distance of 0.1 m similar to experimental distances, we will expect to see VOCs begin to accumulate at the receiver plant in less than 30 min. We anticipate the diffusion process modelled is largely similar for different VOCs emitted by plants, and the transport processes governing VOC transport through the stomata, cuticle, air spaces, and cell wall are also non‐specific (Widhalm et al. 2023). The experimental data then suggests the presence of unique, plant species‐dependent VOC emission mixtures and VOC‐specific recognition mechanisms. VOC perception at the plasma membrane is believed to be comprised of both non‐specific and specific perception mechanisms, including absorption into the plasma membrane and recognition by specific sites that enable VOC transport into the cytosol or trigger changes in membrane potential (Kessler et al. 2023). Based on this, we constructed a simple kinetic model based on reversible binding mechanisms to describe the H_2_O_2_ and SA generation data.

First, we modelled the general recognition of a VOC as reversible ligand binding:

$$\text{VOC}\;+\;\text{Recognition}\;\text{Site}\overset{K_{x}}{⟷}\text{bound}\;\text{VOC}\overset{k_{y}}{⟶}\text{signal}$$

Assuming this reversible binding mechanism reaches quasi‐equilibrium rapidly upon VOC arrival, we estimated the generation rate of downstream signal (i.e., H_2_O_2_ or SA) as proportional to the fraction of bound VOC recognition site, represented by a Hill equation:

$$\frac{d[\text{signal}]}{\mathrm{dt}}\propto \;k\frac{{\left[\text{VOC}\right]}^{n}}{K_{D}^{n}+{\left[\text{VOC}\right]}^{n}}$$

where $K_{D}$ is the dissociation constant for a given recognition site and n is the Hill coefficient, which accounts for cooperativity. Due to lack of upstream kinetic data, we assume high Hill coefficients to approximate threshold‐like activations in the mechanisms discussed below. $K_{D}$, the sensitivity of recognition for a given VOC, thus dictates the onset of downstream signalling.

Using these concepts, we modelled the following signals at the receiver plant for spearmint intercropping (Figure 2b):

$$\frac{d\left[H_{2}O_{2}\right]}{\mathrm{dt}}\propto \;k_{f}\frac{{\left[\text{VOC}\right]}^{n}}{K_{D,\text{VOC\_}H_{2}O_{2}}^{n}+{\left[\text{VOC}\right]}^{n}}-k_{f_{\mathrm{red}}}\frac{{\left[\mathrm{SA}\right]}^{n}}{K_{D,SA_{\mathrm{neg}}}^{n}+{\left[\mathrm{SA}\right]}^{n}},$$

$$\frac{d\left[\mathrm{SA}\right]}{\mathrm{dt}}\propto \;k_{1}\frac{{\left[\text{VOC}\right]}^{n}}{K_{D,\text{VOC\_}\mathrm{SA}}^{n}+{\left[\text{VOC}\right]}^{n}}-k_{2}\left[\mathrm{SA}\right].$$

When enough VOC accumulates, at $K_{D,\text{VOC}{\mathrm{\_H}}_{2}O_{2}}=1.05µM$ and $K_{D,\text{VOC}\mathrm{\_SA}}=$ 0.50 µM, the initial SA and H_2_O_2_ signals are generated. When enough SA accumulates at $K_{D,{\mathrm{SA}}_{\mathrm{neg}}}=3.35µM$, a negative feedback signal reduces the H_2_O_2_ generation rate. SA is known to play a vital role in ROS homoeostasis in plants acting as both pro‐oxidant and antioxidant (Saleem et al. 2021). Detailed kinetic parameters and threshold $K_{D}\;$values are summarized in Supplementary Table 1. Under certain stress conditions, SA is shown to promote ROS scavenging thereby reducing H_2_O_2_ levels (Herrera‐Vásquez et al. 2015; Chang et al. 2024). Hence, we have included a first‐order decay of SA to account for this SA feedback signal. Here, [VOC] is treated as an aggregate signal combining many individual VOCs and recognition mechanisms. The modelled data were found to fit well with the observed experimental time curves for spearmint intercropping with pak choy, suggesting the feasibility of the mechanisms.

### 2.2 ROS of Distinct Sources Generated Upon VOC Perception

It is known that upon stress perception, ROS can be generated within various cell organelles through both enzymatic and non‐enzymatic pathways. Plasma membrane‐localized NADPH oxidases are the most studied enzyme that is involved in ROS production in apoplast. To discern if the intracellular generation of ROS in the receiver pak choy differs when exposed to VOCs from sweet basil and spearmint, we used diphenyleneiodonium (DPI), an NADPH oxidase inhibitor. As control, leaves were treated with 4% DMSO in sterile water (blank) as DMSO was the solvent used for preparation of DPI stock solution, which was then diluted in sterile water. To investigate whether infiltration of 4% DMSO in sterile water has any effect on nanosensor quenching response, pak choy plants infiltrated with H_2_O_2_ nanosensor were treated with the 4% DMSO in sterile water, before the sweet basil emitter plants were placed in proximity. Here, the receiver pak choy plants showed similar ROS production as non‐treated plants placed with sweet basil companion plants (Figure 3a). However, with DPI treatment, changes in ROS dynamics were observed. When sweet basil was used as an emitter, DPI treatment largely inhibited the fluorescence quenching response of the ROS sensor, indicating a lack of ROS generation in the receiver pak choy plants (Figure 3b,d). This suggests that ROS generated in pak choy when exposed to sweet basil VOCs is largely produced by NADPH oxidase in the apoplast. With spearmint as emitter, adding the DPI treatment did not cause significant deviation of the H_2_O_2_ response in the receiver pak choy plants (Figure 3c,e), with similar onsets of ROS production observed for both DPI‐treated and non‐treated plants. This suggests that ROS generation in receiver pak choy plants with spearmint as emitter, is mainly independent of NADPH oxidase.

**Figure 3:**
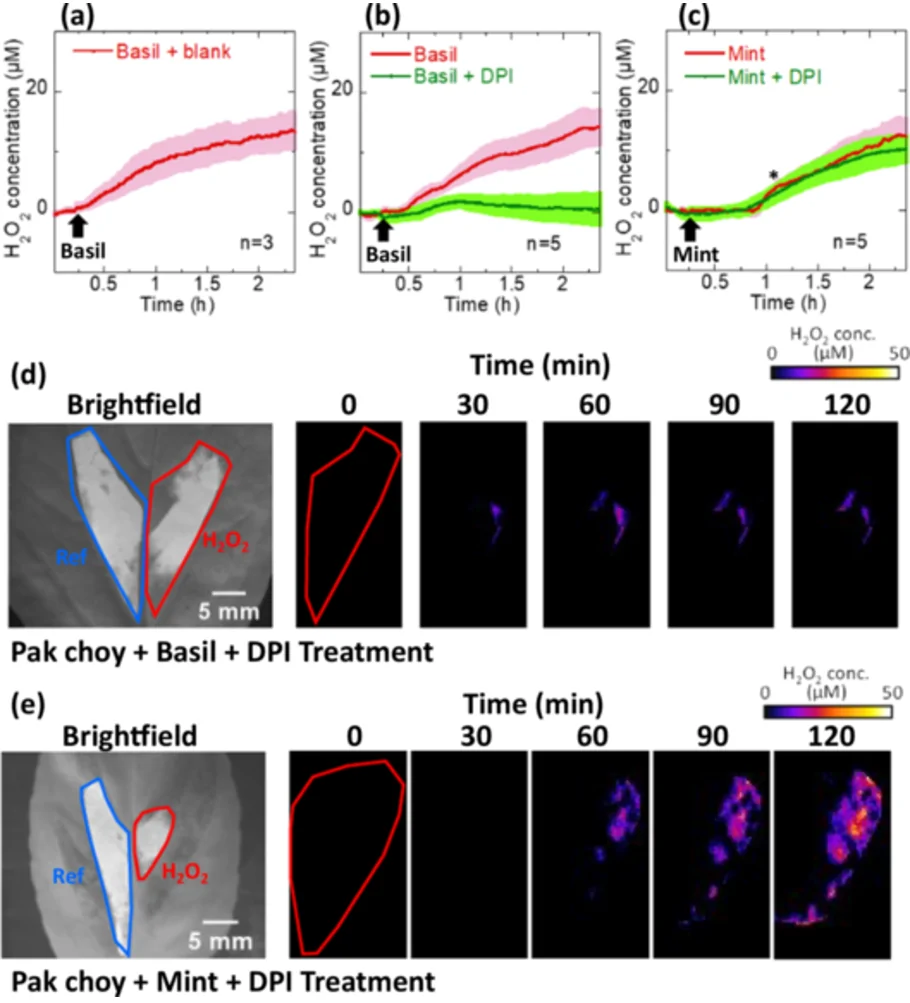
(a) Time‐plots of H_2_O_2_ concentration change derived from nanosensor experiments of (a) pak choy treated with a control solution (4% DMSO) and intercropped with 2 sweet basil plants at *t* = 15 min (black arrow), (b) DPI‐treated (green) and non‐treated (red) pak choy that were intercropped with 2 sweet basil plants at *t* = 15 min (black arrow), (c) DPI‐treated (green) and non‐treated (red) pak choy that were intercropped with 2 spearmint plants at *t* = 15 min (black arrow). Shaded regions represent standard error across 3 to 5 independent biological replicates. Asterisk (*) represents the timepoint at which there is a change in the rate of H_2_O_2_ generation from spearmint intercropping, due to the negative feedback from SA accumulation. Representative brightfield images and H_2_O_2_ concentration maps of DPI‐treated pak choy plant subjected to intercropping with (d) sweet basil and (e) spearmint companion plants at 30‐min intervals. [Color figure can be viewed at wileyonlinelibrary.com]

Ca^2+^ binding is known to activate NADPH oxidase enzyme activity to generate apoplastic ROS (Ogasawara et al. 2008; Kimura et al. 2012; Oda et al. 2010). Therefore, for the sweet basil intercropping kinetic model, we postulated that there is a cytosolic Ca^2+^ influx‐dependent mechanism for activating of NADPH oxidase (Zhang et al. 2025) that generates ROS in receiver pak choy plants. Aratani et al. 2023 demonstrated that certain VOC exposures induce cytosolic Ca^2+^ influx in Arabidopsis while others do not (Aratani et al. 2023). This suggests that VOC‐specific cytosolic Ca^2+^ influx could be key in the different kinetics observed in sweet basil‐ and spearmint‐induced downstream H_2_O_2_ generation. Hence, we modelled the sweet basil intercropping signals (Figure 2c) as

$$\frac{d\left[Ca^{2+}\right]}{\mathrm{dt}}\propto \;k_{C}\frac{{\left[\text{VOC}\right]}^{n}}{K_{D,\text{VOC\_Ca}}^{n}+{\left[\text{VOC}\right]}^{n}},$$

$$\frac{d\left[H_{2}O_{2}\right]}{\mathrm{dt}}\propto \;k_{f}\frac{{\left[{\mathrm{Ca}}^{2+}\right]}^{n}}{K_{D,P}^{n}+{\left[{\mathrm{Ca}}^{2+}\right]}^{n}}-k_{r}\left[H_{2}O_{2}\right],$$

$$\frac{d\left[\mathrm{SA}\right]}{\mathrm{dt}}\propto \;k_{1}\frac{{\left[\text{VOC}\right]}^{n}}{K_{D,\text{VOC\_}\mathrm{SA}}^{n}+{\left[\text{VOC}\right]}^{n}}-k_{2}\left[\mathrm{SA}\right].$$

where Ca^2+^ influx is modelled with a generic hill equation based on VOC concentration ${(K}_{D,\text{VOC\_Ca}})$. Then, H_2_O_2_ is generated as enough Ca^2+^ binds NADPH oxidase (P) at $K_{D,P}=0.08\;µM$ and SA is generated as enough VOC accumulates at $K_{D,\text{VOC}\mathrm{\_SA}}$ = 0.02 µM. H_2_O_2_ and SA both have first‐order decay terms, but SA does not negatively feedback to H_2_O_2_ generation. Ca^2+^ efflux is not explicitly modelled, as it is assumed to be in excess of NADPH oxidase. Despite the relative simplicity of the proposed model, the modelled data provide a reasonably accurate depiction of the experimental ROS and SA concentration–time curves for sweet basil intercropping, which is distinct from spearmint intercropping.

### 2.3 Spearmint and Sweet Basil VOC Emission Profile

To elucidate if the difference in ROS and SA generation in the receiver plant is due to the difference in emitted VOC profile of the plants, the major VOCs emitted by spearmint, sweet basil plants and pak choy plants were identified using GC‐MS SPME. The GC‐MS analysis revealed that the three plants shared some common VOCs, as well as VOCs that are unique to each of them (Supplementary Table 2). To identify if individual VOCs can generate distinct SA and ROS signalling, or a VOC blend is required, pak choy plants infiltrated with both ROS and SA nanosensors were exposed to a single VOC disc (Supplementary Figure 2a), containing either eugenol or carvone that are unique to sweet basil or spearmint plants, respectively, according to the GC‐MS SPME results. With exposure to eugenol, simultaneous generation of H_2_O_2_ and SA accumulation were observed in the receiver pak choy plant (Supplementary Figure 2b), with similar onsets of production to when sweet basil companion plants were used (Supplementary Figure 2c). The amount of ROS and SA produced by the end of 2 h was 12.1 ± 4.0 and 8.3 ± 2.1 μM, respectively. Hence, comparing eugenol exposure with having sweet basil companion plants, the ROS concentration in receiver pak choy at *t* = 2 h are similar, whereas SA concentration for eugenol exposure is slightly lower than having sweet basil companion plants. When carvone was used, the sensors report a relatively delayed H_2_O_2_ production compared to SA (Supplementary Figure 2d), similar to when spearmint companion plants were used (Supplementary Figure 2e). However, in comparison to having spearmint companion plants, the amount of SA produced at *t* = 2 h was higher at 12.9 ± 1.8 μM and amount of ROS produced was lower at 6.4 ± 1.1 μM when carvone was used. Previous studies have shown that GLVs, such as (*Z*)−3‐hexen‐1‐ol, are emitted in relatively high levels in Brassicaceae vegetables including pak choy (Liu et al. 2018). With exposure to (*Z*)−3‐hexen‐1‐ol, the sensors reported negligible H_2_O_2_ production by the end of 2 h (Supplementary Figure 2f). Interestingly, SA sensor detected a decrease in SA levels due to (*Z*)−3‐hexen‐1‐ol exposure. By *t* = 2 h, the reduction in SA levels is 8.1 ± 1.4 μM. These differences in individual VOC‐induced ROS and SA production could be attributed to the concentration variation and presence of other volatile compounds in the VOC blends emitted by the companion plants. Overall, the above experiments depict that exposure to single VOCs emitted by spearmint and sweet basil plants can trigger generation of ROS and SA with similar kinetics in receiver pak choy plants.

### 2.4 Sweet Basil and Spearmint VOCs Elicit Some Similar but Vastly Different Transcriptional Changes in Pak Choy Plants

To comprehend the effect of sweet basil/spearmint VOCs on the gene expression in pak choy leaves, transcriptome analysis was performed after 2 h of intercropping. Using the same experimental conditions as the sensor experiments, pak choy leaves were harvested after 2 h of exposure to either sweet basil or spearmint plants. As control, leaves from pak choy exposed to pak choy plants were used. Significant DEGs with a log_2_ fold change ≥ 1 and FDR < 0.05 were selected. Following these criteria, a total of 5838 (3538 upregulated and 2300 downregulated) genes were identified in pak choy leaves exposed to sweet basil VOCs (Supplementary Figure 3a). When grown with spearmint, a total of 5931 (3001 upregulated and 2930 downregulated) genes were observed (Supplementary Figure 3b). Gene ontology (GO) terms and Kyoto Encyclopedia of Genes and Genomes (KEGG) pathway functional enrichment were performed to obtain gene function information and biological processes associated with the DEGs.

The top 15 GO terms associated with the upregulated DEGs of pak choy–sweet basil group is shown in Figure 4a. The upregulated biological processes (BP) were mainly involved in response to chemical, carbohydrate and polysaccharide metabolism and response to auxin. The upregulated cellular components (CC) were mainly apoplast, extracellular region, external encapsulating structure organization, cell wall and cell periphery. Among the enriched GO terms in the molecular function (MF) category, the dominant ones were oxidoreductase activity, xyloglucan: xylogluocosyl transferase activity, iron ion binding, tetrapyrrole binding and haem binding. There were only 8 significant GO terms associated with the downregulated DEGs of pak choy–sweet basil group (Figure 4b). The 2 downregulated BP were mainly DNA replication and DNA metabolic process. The 6 downregulated CC were mitochondrial matrix, chromosomal part, intracellular organelle lumen, organelle lumen and membrane‐enclosed lumen and chromosome. No significantly downregulated GO terms were identified in the MF category.

**Figure 4:**
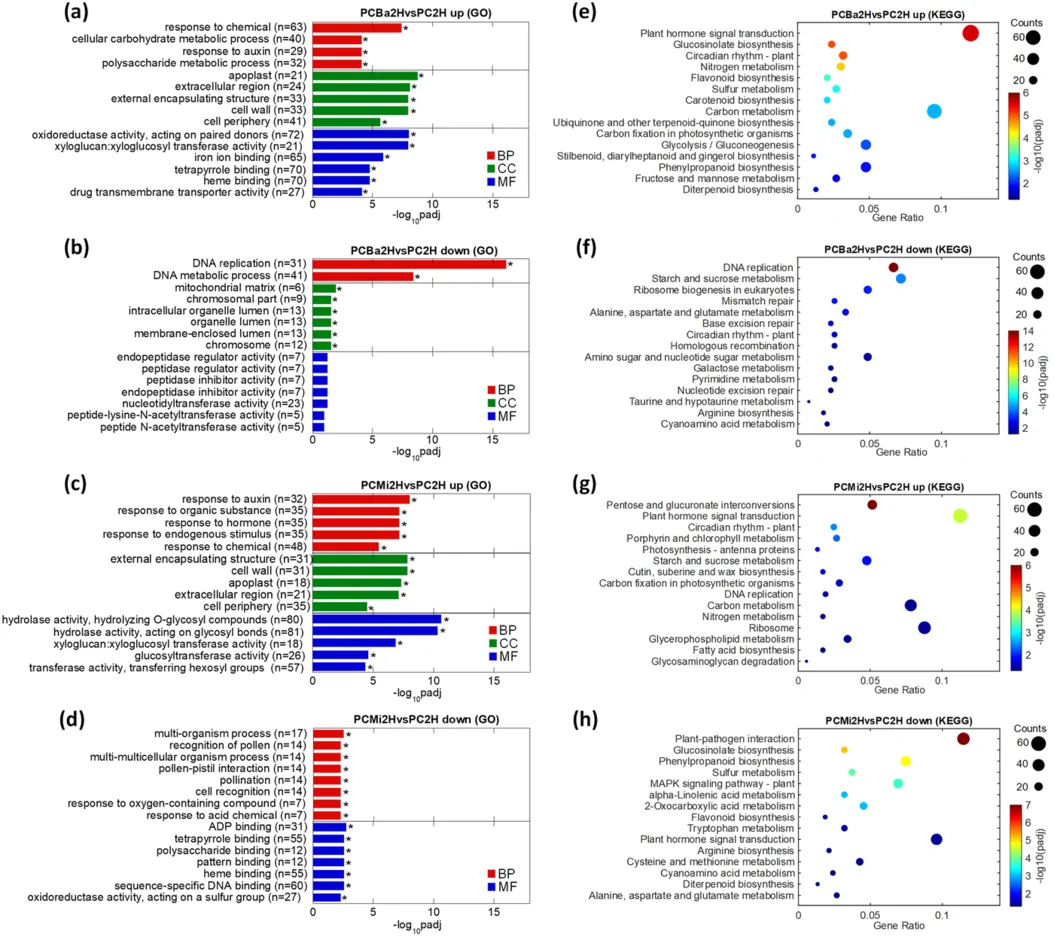
Transcriptome analysis showing GO and KEGG pathway enrichment of differentially expressed genes between monocropped and intercropped pak choy based on RNA‐sequencing data. Top 15 GO terms in (a) upregulated and (b) downregulated DEGs from pak choy–sweet basil intercropping. Top 15 GO terms in (c) upregulated and (d) downregulated DEGs from pak choy–spearmint intercropping. GO terms are categorized into biological processes (BP, red), cellular components (CC, green) and molecular functions (MF, blue). Gene counts (*n*) are listed in parentheses; Top 15 KEGG pathways in (e) upregulated and (f) downregulated DEGs from pak choy–sweet basil intercropping. Top 15 KEGG pathways in (g) upregulated and (h) downregulated DEGs from pak choy–spearmint intercropping. Size of data bubbles correspond to gene counts. Colour scale represents $-\log_{10}\left(\mathrm{padj}\right)$ with minimum colour threshold set at $-\log_{10}\left(\mathrm{padj}\right)=1.3$, which indicates significantly differentially expressed genes. KEGG pathways with $-\log_{10}\left(\mathrm{padj}\right)<1.3$ are indicated as dark blue data bubbles. [Color figure can be viewed at wileyonlinelibrary.com]

Figure 4c shows the top 15 GO terms associated with the upregulated DEGs in the pak choy–spearmint group. The enriched BP were mainly responsive to auxin, organic substance, hormone, endogenous stimuli and chemical. The upregulated CC were external encapsulating structure organization, cell wall, apoplast, extracellular region, and cell periphery. In the MF category, hydrolase activity, xyloglucan:xyloglucosyl transferase activity and glucosyltransferase activity were most enriched. Of the top 15 significant GO terms associated with the downregulated DEGs of pak choy–spearmint group, the dominant group in the BP category was multiorganism process, pollination and cell recognition process (Figure 4d). In MF category, ADP binding, tetrapyrrole binding, polysaccharide binding, pattern binding, haem binding and oxidoreductase activity were dominant. There were no significant downregulated GO terms in CC category. Overall, there were some shared GO terms associated with upregulated DEGs in both groups like response to auxin, response to chemical, apoplast, external encapsulating structure organization and cell wall and cell periphery. Some GO terms associated with upregulated DEGs of the pak choy–sweet basil group were found associated with downregulated DEGs of pak choy–spearmint group like tetrapyrrole binding, haem binding, and oxidoreductase activity—acting on paired donors.

The top 15 significantly enriched KEGG pathways from the upregulated DEGs of pak choy–sweet basil group are shown in Figure 4e, which includes many key primary metabolic pathways involved in plant growth and development like plant hormone signal transduction, carbon metabolism, carbon fixation, nitrogen and sulphur metabolism and glycolysis and gluconeogenesis. Interestingly many secondary metabolic pathways like glucosinolates, carotenoid, flavonoid and phenylpropanoid biosynthesis pathways were also found to be enriched indicating significant biochemical alterations in pak choy leaves after exposure to sweet basil VOCs. The downregulated DEGs were significantly enriched in 7 pathways of which DNA replication, starch and sucrose metabolism are the dominant ones (Figure 4f).

The KEGG pathway analysis of the pak choy–spearmint group demonstrated that the upregulated DEGs were significantly enriched in only four different pathways, namely pentose and glucuronate interconversions, plant hormone signal transduction, circadian rhythm, porphyrin and chlorophyll metabolism (Figure 4g). The plant hormone signal transduction and circadian rhythm pathways are shared between the two groups, suggesting that both sweet basil and spearmint VOCs can lead to alterations in these pathways.

Nanosensor experiments revealed the production of SA within 2 h in receiver pak choy with both the emitters. We identified transcripts of genes involved in SA biosynthesis, including isochorismate synthase, phenylalanine ammonia‐lyase 1 and 4, and chorismate mutase, which were upregulated in pak choy when exposed to sweet basil VOCs. These genes were not differentially expressed in pak choy–spearmint group at the 2 h timepoint. LC‐MS analysis showed (Figure 2g) that the amount of SA produced is more in pak choy leaves exposed to sweet basil VOCs at 2 h in comparison to pak choy exposed to spearmint. The above expression pattern of these genes supports this observation, where a continued upregulation of SA biosynthesis genes is observed in the pak choy–sweet basil group, but not in the pak choy–spearmint group.

Based on the RNA‐seq data, another dominant hormone‐related category was transcripts associated with auxin signalling. The GO term ‘response to auxin’ is enriched in both groups and auxin plays an important role in plant growth and development. Transcripts for different auxin‐related proteins like *small auxin upregulated RNA* (SAUR) SAUR21, auxin/indole acetic acid (Aux/IAA) proteins IAA17 and IAA7 and various auxin efflux carrier components were found upregulated in both categories. With sweet basil companion plants, additional transcripts related to auxin biosynthesis and signalling were found to be upregulated like tryptophan aminotransferase‐related protein 4, indole‐3‐pyruvate monooxygenase YUCCA8, indole‐3‐pyruvate monooxygenase YUCCA1, indole‐3‐acetaldehyde oxidase, nitrilase 2 and auxin‐responsive protein SAUR32. To confirm whether the expression of these genes indicates increased auxin production, we performed LC‐MS analysis to determine the IAA content in the leaves at 2 h post exposure to VOCs in pak choy (Supplementary Figure 4). An increase in IAA levels was observed in pak choy leaves with both emitter plants, but the increase was more with sweet basil as emitter. This suggests that the VOCs from sweet basil stimulate a stronger auxin biosynthesis in pak choy, as compared to spearmint VOCs.

The circadian clock is widely involved in the temporal control of plant development, physiology, and environmental response (Sanchez and Kay 2016). In the circadian rhythm pathway, transcripts encoding for two‐component response regulator‐like APRR9, SPA (suppressor of phyA‐105), elongated hypocotyl5 (HY5), *Revielle* (*RVE*), and cyclic dof factor 1‐like were observed but the upregulation of these genes was more pronounced in the pak choy–sweet basil group. In addition, genes like late elongated hypocotyl (LHY), *night light–inducible and clock‐regulated2* (*LNK2*) were exclusively upregulated in the pak choy–sweet basil group. Studies have shown that circadian clock regulates auxin signalling and responses (Covington and Harmer 2007; Srivastava et al. 2019).

The downregulated DEGs from the pak choy–spearmint group were significantly enriched in 7 different pathways as shown in Figure 4h, which included glucosinolates biosynthesis, sulphur metabolism and phenylpropanoid biosynthesis. These pathways are associated with the upregulated DEGs in pak choy–sweet basil group. Overall, the transcriptome analysis suggests that exposure to sweet basil or spearmint VOCs induces few common but largely distinct modifications in the receiver pak choy plants. The common VOCs present in both the emitter plants can be the reason for the similar effects observed in the transcriptome data. The expression levels of selected significant DEGs from major pathways were validated by RT‐qPCR (Supplementary Table 3).

### 2.5 Companion Planting With Sweet Basil Induced an Increase in Biomass

To determine whether the VOC‐induced signalling in pak choy has any phenotypic effect, pak choy seedlings were grown alongside either spearmint or sweet basil plants in separate pots in the same ratio as the sensor experiments (1 pak choy: 2 sweet basil/spearmint) in the box for 5 weeks, and its growth and development were monitored (Figure 1a). Pak choy plants grown alongside sweet basil showed a significant increase in both shoot (Figure 5a,b) and root (Figure 5c,d) biomass. The roots were more branched, denser and bigger. However, pak choy plants grown with spearmint did not present any increase in biomass. We further repeated this experiment in a small growth chamber, but with a different ratio of 3 pak choy: either 1 sweet basil or spearmint, to mimic an intercrop setting. The selected ratio gave a similar increase in biomass of pak choy (Supplementary Figure 5a), as observed with the ratio used for the box experiments. Intercropping with spearmint displayed no increase in biomass when compared to control monocropped in the same ratio. In summary, the sweet basil VOCs promoted growth in receiver pak choy plants. Since eugenol, a VOC specifically emitted by sweet basil, was able to induce similar SA and ROS signalling, we analysed its effect on pak choy upon direct exposure. Pak choy plants were grown in a box setting, and exposed to 100 µM of eugenol for 4 weeks and their growth was subsequently examined (Supplementary Figure 5b,c). An increase in shoot and root biomass was observed in pak choy plants, but the increase was much smaller compared to what was observed when sweet basil plants were used. Hence, further optimization of the eugenol concentration, as well as extent of exposure, is required to achieve a comparable increase in biomass, similar to pak choy intercropping with sweet basil.

**Figure 5:**
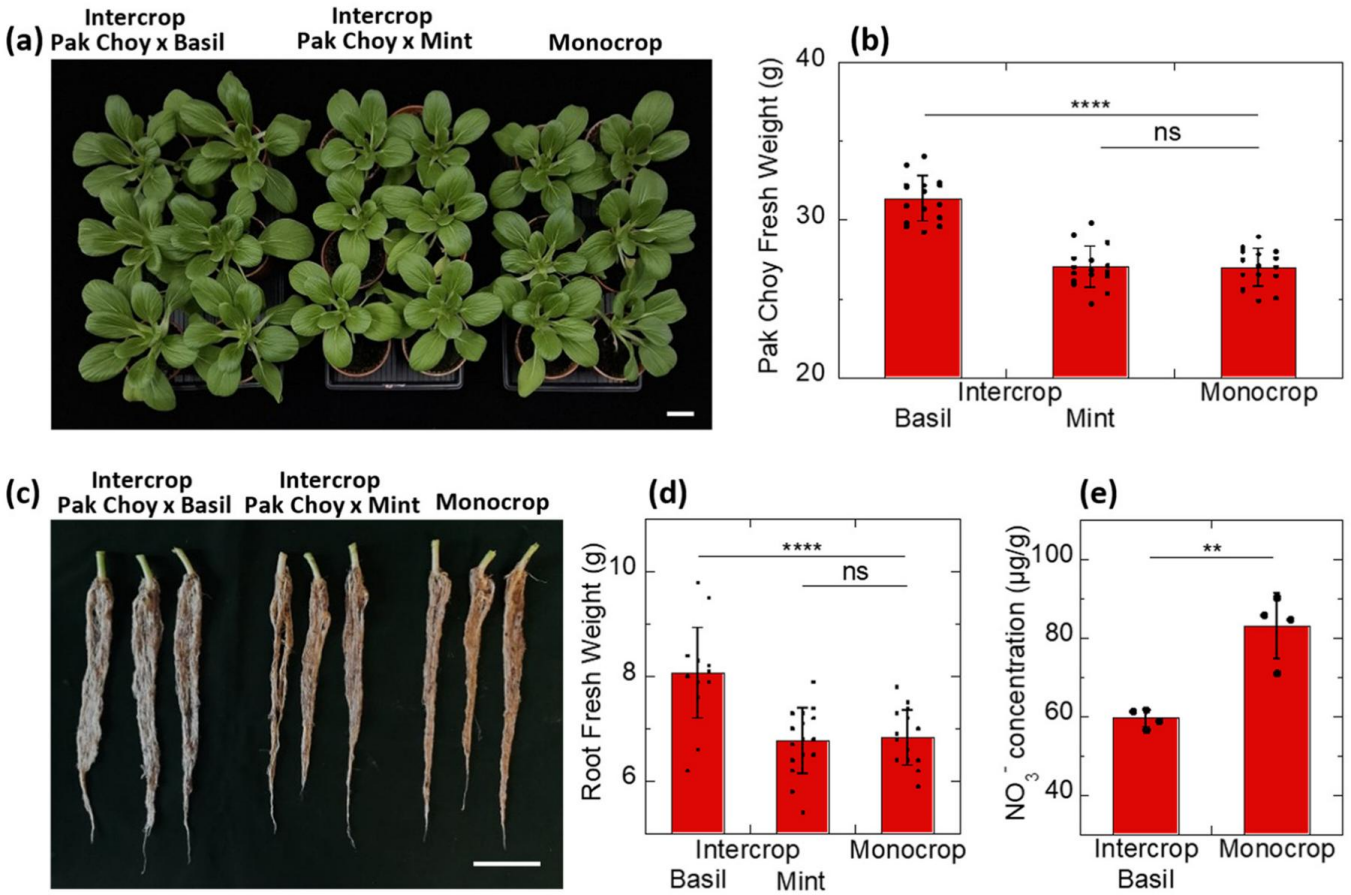
Enhanced (a, b) shoot biomass and (c, d) root biomass observed in pak choy when grown at 1:2 ratio next to sweet basil, compared to spearmint and monocropped with pak choy. Scale bars in (a, c) represent 5 cm. Bar graphs show the mean biomass values with error bars representing standard deviations from *n* = 16 independent biological replicates. Dots represent each data points. Two‐tailed unpaired *t* test, **p < 0.01, ****p < 0.0001; (e) reduced amount of nitrates when pak choy plants were intercropped with sweet basil for 5 weeks at 1:2 ratio. Bar graph show the mean values with error bars representing standard deviations from independent biological replicates (*n* = 4). Individual data points are shown as dots. Two‐tailed unpaired *t* test, **p < 0.01. [Color figure can be viewed at wileyonlinelibrary.com]

While numerous studies demonstrate that VOCs can enhance plant growth and health, especially bacterial/fungal VOCs, their mechanisms of action remain poorly elucidated. We examined the transcriptome data further to elucidate the molecular mechanisms behind the increase in biomass observed in pak choy when exposed to sweet basil VOCs. KEGG pathway analysis of pak choy–sweet basil group showed a very significant enrichment of primary metabolic pathways like carbon metabolism, carbon fixation, nitrogen and sulphur metabolism, glycolysis and gluconeogenesis. All these pathways can greatly influence plant growth and yield especially carbon and nitrogen metabolism, which are also closely linked to key fundamental metabolic processes. Transcripts encoding enzymes of Calvin‐Benson cycle (CBC), which regulate the photosynthetic efficiency of plants were found to be upregulated. These included ribulose bisphosphate carboxylase/oxygenase activase, ribulose bisphosphate carboxylase small chain, Phosphoribulokinase, phosphoenolpyruvate carboxylase 2, glyceraldehyde‐3‐phosphate dehydrogenase A. Altering the activity of enzymes in the CBC in plants has been demonstrated to directly influence carbon assimilation and plant growth (Muhie 2022). We looked for similar transcripts in the pak choy–spearmint group and found that ribulose bisphosphate carboxylase/oxygenase activase is upregulated but to a smaller degree.

Nitrogen is an important macronutrient for plants and is a key component of proteins, enzymes and chlorophyll. An increased nitrogen use efficiency (NUE) is associated with higher yield and quality of crops (Peinado‐Torrubia et al. 2023). DEGs associated with nitrogen metabolism pathway included key genes involved in nitrogen assimilation like nitrate reductase (NR), nitrite reductase (NiR), and cytosolic glutamine synthetase cytosolic isozyme 1‐2 (GS), all of which were upregulated. Nitrate reductase (NR) and nitrite reductase (NiR), were found downregulated in pak choy–spearmint group. Overexpression of NiR is associated with reduced nitrate levels in leaves indicative of higher nitrogen assimilation (Davenport et al. 2015; Curtis et al. 1999). In view of this, we measured the nitrate levels in pak choy leaves and found that its concentration was significantly reduced (Figure 5e). A critical coordination between carbon and nitrogen metabolism is essential for optimal plant growth, and RNA‐seq analysis indicates that both pathways are positively influenced in pak choy exposure to sweet basil VOCs.

Sulphur is a vital nutrient needed for plant growth and development. DEG associated with sulphur metabolism included transcripts encoding ATP sulfurylases, serine acetyltransferase (mitochondrial), bifunctional cystathionine gamma‐lyase/cysteine synthase, assimilatory sulphite reductase (ferredoxin), all of which are involved in the assimilation of sulphate to cysteine. In plants, cysteine serves as the sulphur donor and the precursor for the formation of numerous organic sulphur compounds (Narayan et al. 2023). Transcript encoding adenylyl‐sulphate kinase which catalyses the synthesis of activated sulphate that is required to produce secondary metabolites like glucosinolates was also upregulated. In plants, there is a known interaction between sulphur and nitrogen metabolism influencing each other's assimilation and accumulation of secondary metabolites (Carfagna et al. 2011; Miao et al. 2018). With spearmint as emitter, sulphur metabolism pathway was downregulated.

Glycolysis and gluconeogenesis pathway are key energy‐providing pathways influencing plant growth. Significant DEGs upregulated under this category included genes encoding for fructose‐1,6‐bisphosphate aldolase, pyruvate dehydrogenase E1 component, aldehyde dehydrogenase family 3‐member, hexokinase‐3‐like, pyrophosphate‐fructose 6‐phosphate 1‐phosphotransferase subunit alpha 2. Overexpression of fructose‐1,6‐bisphosphate aldolase has been shown to enhance plant growth and biomass (Uematsu et al. 2012). In conclusion, transcriptome analysis reveals that exposure to sweet basil VOCs alters key primary metabolic pathways in pak choy, leading to enhanced overall plant growth.

### 2.6 Sweet Basil Volatiles Promote Secondary Metabolite Accumulation in Pak Choy

KEGG annotation indicated upregulation of secondary metabolic pathways in pak choy exposed to sweet basil VOCs, namely glucosinolates, flavonoid and carotenoid biosynthesis. Glucosinolates are sulphur‐containing phytochemicals mainly found in Brassica vegetables like pak choy. Transcripts for genes involved in glucosinolates biosynthesis like *S*‐alkyl‐thiohydroximate lyase SUR1, cytosolic sulfotransferases and cytochrome P450 83A1‐like, have significant higher expression levels. DEGs involved in flavonoid and carotenoid metabolism included transcripts encoding for flavonol synthase/flavanone 3‐hydroxylase, caffeoyl‐CoA O‐methyltransferase, *trans*‐cinnamate 4‐monooxygenase‐like, chalcone synthase 3‐like, shikimate *O*‐hydroxycinnamoyl transferase, phytoene synthase, beta‐carotene hydroxylase, beta‐carotene isomerase and zeaxanthin epoxidase. In view of the above, we measured the total amount of these phytochemicals in pak choy leaves upon harvest. Examination revealed a notable rise in total flavonoids (49%), carotenoids (22%) and glucosinolates (60%) leading to a substantial improvement in the nutraceutical quality of pak choy (Figure 6a–c). These results suggest that exposure to sweet basil VOCs markedly elevates secondary metabolite levels in pak choy.

**Figure 6:**
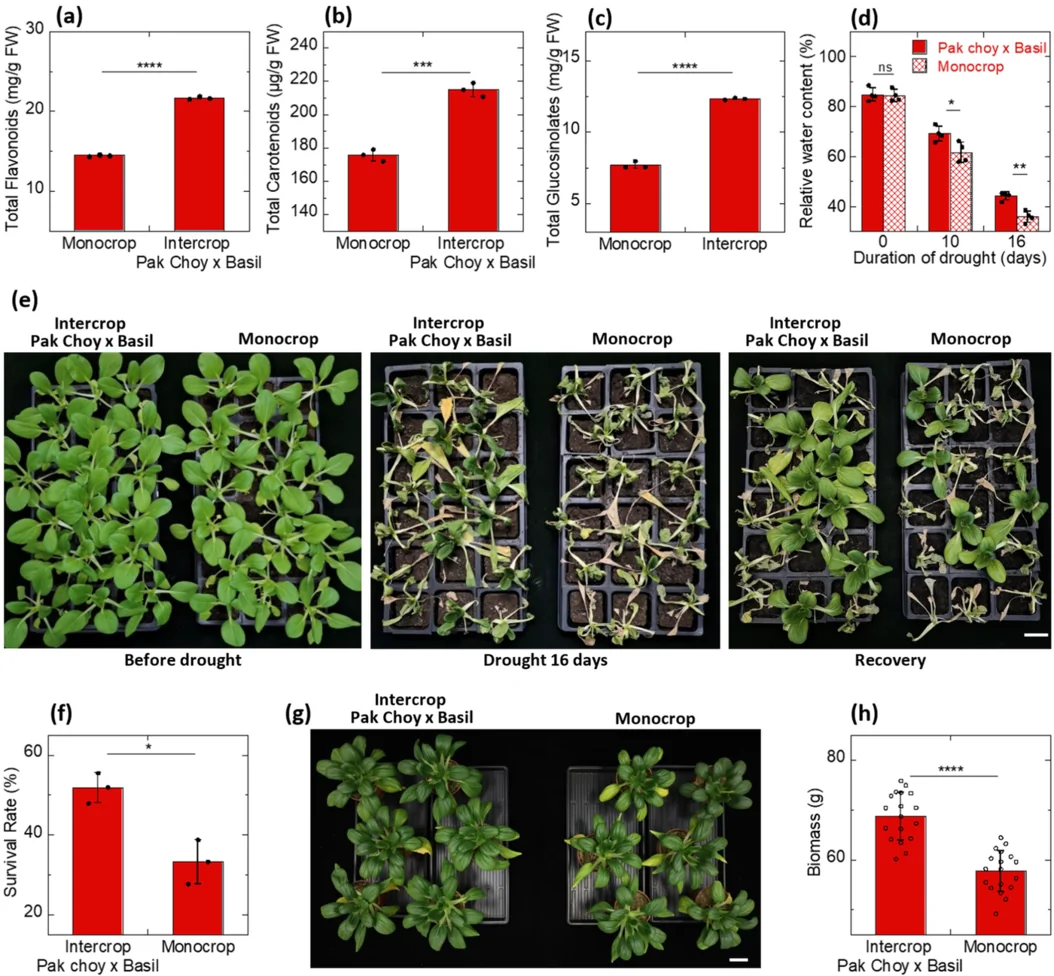
Bar graphs show increase in mean values of (a) total flavonoids (b) total carotenoids (c) total glucosinolates when pak choy plants are intercropped with sweet basil at 1:2 ratio for 5 weeks (*n* = 3). Dots represent each data points. Error bars represent standard deviations from *n* = 3 independent biological replicates. (d) Relative water content in pak choy plants when intercropped with sweet basil (block) and monocropped (diagonal stripes), under drought stress for 0, 10 and 16 days. Bar graphs show mean values with dots representing each experimental data point and error bars representing standard deviations from *n* = 4 independent biological replicates. (e) Photographs of pak choy plants intercropped with sweet basil and monocropped, undergoing drought stress and subsequent recovery. Scale bar: 5 cm. (f) Bar graph showing increased survival percentage of pak choy intercropped with sweet basil compared to monocropped (*n* = 3). Dots represent individual experimental sets which each comprises 18 biological replicates, and error bars represent standard deviation from the experimental sets. (g) Photographs of pak choy plants intercropped with sweet basil and monocropped, under nitrate deficiency (1.5 mM) conditions. Scale bar: 5 cm. (h) Bar graph showing increase in average biomass of pak choy plants intercropped with sweet basil under low nitrate conditions. Dots represent individual data points while error bars represent standard deviation from *n* = 18 biological replicates. Two‐tailed unpaired *t* test, *p < 0.05, **p < 0.01, ***p < 0.001, ****p < 0.0001. [Color figure can be viewed at wileyonlinelibrary.com]

### 2.7 Drought and Nitrogen Stress Resistance Observed in Pak Choy When Grown Next to Sweet Basil

Drought response is closely linked to Nitrogen and Carbon metabolism in plants (He et al. 2022; Pandey et al. 2022; Cui et al. 2019). Production of secondary metabolites like carotenoid, flavonoids and glucosinolates is also known to provide protection against abiotic and biotic stress including drought conditions (Salehin et al. 2019; Yadav et al. 2021; Ghasemi et al. 2023). Given the increase in secondary metabolite levels and root biomass, we examined the drought resilience of pak choy when grown with sweet basil. Drought resilience is defined as the ability of a plant to tolerate water stress and recover upon re‐watering. Drought response was analysed on pak choy plants grown in small growth chamber with sweet basil in a 3:1 ratio and control monocropped pak choy plants. After 3 weeks of growth, water was withheld for 16 days. Leaf relative water content (RWC) was measured on Days 0, 10 and 16. Intercropped pak choy showed significantly increased RWC compared to monocropped pak choy plants (Figure 6d). Upon re‐watering on day 16, survival percentage of pak choy plants intercropped with sweet basil increased by 25% (Figure 6e,f).

Changes in root architecture, in terms of increased root density and root surface area are associated with enhanced N uptake in plants. Additionally, changes in enzymes related to nitrogen metabolism like overexpression of nitrate reductase, nitrite reductase are associated with increased NUE. An improved NUE can enhance crop productivity with less nitrogen fertilizer input (Lebedev et al. 2021; Govindasamy et al. 2023). Based on the observed changes in the transcriptome and increased root biomass in Pak Choy exposed to sweet basil VOCs, we investigated whether these plants exhibit enhanced tolerance to low nitrogen conditions. To accurately control the nitrogen input, we grew the plants in vermiculite, which is a nutrient‐free soil, and added Hoagland solution containing decreasing concentrations of nitrate from 2.5, 1.5, 1, 0.5 mM, to no nitrate, and checked the effect on pak choy biomass. We observed a drastic difference in pak choy biomass when grown under 1.5 mM nitrate concentration and below (Supplementary Figure 6). We then selected the 1.5 mM concentration to perform the nitrate tolerance studies. Here, pak choy plants were grown in small growth chambers with either sweet basil (3: 1 ratio) or with pak choy. After 5 week of growth, the plants were harvested and their biomass was measured (Figure 6g). Compared to monocropped pak choy, pak choys intercropped with sweet basil displayed approximately 19% higher biomass when grown under 1.5 mM nitrate (Figure 6h). In summary, plants exposed to sweet basil VOCs exhibited improved tolerance to water stress and low nitrogen conditions.

## 3 Discussion

Increasing evidence indicates VOCs can function as chemical signals transmitted from emitting plants to neighbouring eavesdropping plants, where the perceived VOCs can affect its gene regulation, metabolism, phenotype and stress responses (Loreto and D'Auria 2022). However, the primary events associated in this subtle plant‐to‐plant communication, especially, how receiving plants sense and respond to distinct VOCs remains predominantly unclear. In this study, we used multiplexed nanosensors to visualize in real‐time, the generation of H_2_O_2_ and SA in the leaves of pak choy plants in response to constitutively‐emitted VOCs from different neighbouring plants, namely spearmint, sweet basil and pak choy, which emit different blend of volatiles. Sweet basil and spearmint VOCs were able to generate H_2_O_2_ and SA in receiver pak choy plant, but with distinct dynamics that can be captured within 2 h of intercropping. Pak choy plants as emitters failed to trigger either H_2_O_2_ or SA production in receiver pak choy plant within the monitored time. Both spearmint and sweet basil VOCs can activate SA production in receiver pak choy plants within 30 min. However, by 2 h, significantly higher SA levels were observed in plants exposed to sweet basil VOCs compared to those exposed to spearmint VOCs. Sweet basil VOCs triggered earlier H_2_O_2_ generation within 30 min, whereas spearmint VOCs triggered later H_2_O_2_ generation by 1 h. By 2 h, similar amounts of H_2_O_2_ are observed in receiver pak choy exposed to both emitter plants. Exposure to sweet basil VOCs induced similar amounts of H_2_O_2_ and SA production in receiver pak choy, while exposure to spearmint VOCs generates more H_2_O_2_ than SA by 2 h. This demonstrates that the temporal response of receiver plants' to airborne VOCs is both rapid and dependent on the identity of neighbouring plants, highlighting the intricacies of plant‐to‐plant communication.

Exposure to single volatiles like eugenol and carvone, which are specific to sweet basil and spearmint, respectively, were able to stimulate the production of H_2_O_2_ and SA at similar onsets but varying concentrations, compared with exposure to the companion plants. Exposure to (*Z*)‐3‐hexen‐1‐ol, a GLV molecule emitted by pak choy, did not trigger ROS production and seems to reduce endogenous SA levels. GLVs like (*Z*)‐3‐hexen‐1‐ol are generally produced in response to wounding in plants. Several studies have shown that exposure to GLVs results in the activation of wound‐ and herbivore‐induced defence response via the generation of JA (Aratani et al. 2023; Engelberth et al. 2004; KOST and HEIL 2006; Scala et al. 2013). In plants, JA and SA are typically known to act antagonistically (Zhang et al. 2023). The decrease in SA observed in pak choy after exposure to (*Z*)‐3‐hexen‐1‐ol can possibly be attributed to the generation of JA, which suppresses SA production. Evidence suggests that the components of the VOC blend and their ratios are responsible for generating the specific response in the receiver plant (Fitzky et al. 2021; Ueda et al. 2012). Additionally, the concentration of the pure VOCs used in this study does not mimic the actual amount of VOC being naturally emitted by the companion plant. These could be potential reasons for the variations in H_2_O_2_ and SA generation in receiver pak choy when exposed to single VOCs, rather than VOC blends emitted by the companion plants. Nonetheless, our study demonstrates that both single or blended VOCs can alter ROS and SA levels in pak choy leaves.

By changing the VOC components and blend ratios, plants can create specific messages for communication. Companion planting with sweet basil led to increased biomass in pak choy. No increase in biomass was observed when pak choy was grown alongside spearmint. Nanosensors revealed that VOCs from both aromatic plants lead to the early production of ROS and SA. Reactive oxygen species (ROS) such as H_2_O_2_ are key signalling molecules regulating plant growth and responses to external stimuli (Huang et al. 2019; Tsukagoshi et al. 2010; Zeng et al. 2017). External stimuli can result in the generation of ROS in different cell organelles disrupting the cellular homoeostasis of ROS and generating distinct stress‐specific ROS signature in cells. ROS signalling integrates with other important stress‐response signal transduction pathways and hormones to orchestrate specific stress responses. Therefore, depending on where ROS is produced in a cell in response to an external stimulus, it can activate different signal transduction pathways (Zafra et al. 2010; Mittler et al. 2022; Devireddy et al. 2021). The dominant source of apoplastic ROS is the plasma membrane NADPH oxidases, also named as respiratory burst oxidase homologs (RBOHs). Apoplastic ROS is known to play a vital role in plant development and stress response (Podgórska et al. 2017; Chapman et al. 2019). The H_2_O_2_ nanosensor results illustrate that treatment of pak choy with DPI had a notable suppression of H_2_O_2_ when sweet basil was the emitting plant, as opposed to spearmint. This suggests that the VOCs from sweet basil play a major role in stimulating apoplastic H_2_O_2_. Spearmint VOCs most likely activates H_2_O_2_ in different cellular compartments, like chloroplast or peroxisomes, where H_2_O_2_ accumulation is also known to occur upon external stimuli. The observed differences in the site of origin of ROS in Pak Choy in response to sweet basil or spearmint VOC stimuli may activate distinct signal transduction pathways, leading to different physiological outcomes. These experimental insights allowed us to formulate a kinetics model that effectively captures the unique temporal features of ROS and SA generation induced by spearmint and sweet basil companion plants, respectively, forming the basis of elucidating the different VOC‐mediated signal transduction pathways induced at the pak choy receiver plant. Unlike previous transport‐only models, our model incorporates a physiological VOC concentration threshold. Downstream signalling is triggered only when the internal VOC concentration exceeds this threshold, accounting for the variable ROS and SA signalling delays observed in the receiver pak choy plants intercropped with sweet basil and spearmint emitters.

SA is an important phytohormone that plays a key role in plant stress responses. SA can act as both pro‐oxidant and antioxidant, regulating ROS homoeostasis in plants. Research indicate a significant interaction between ROS and SA during biotic responses which is influenced by the source of ROS and the type of pathogen (Lukan and Coll 2022; Torres et al. 2005). The role of SA in influencing plant growth and development under normal conditions, when compared to its function in plant stress responses, is far less understood. Changes in endogenous SA levels are associated with altered growth phenotypes. In Arabidopsis, mutants with higher amounts of SA showed stunted growth, while those with reduced SA exhibited increased shoot biomass (Fujikura et al. 2020; Rate et al. 1999; Miura et al. 2010; Abreu and Munne‐Bosch 2009). However, rice mutants with lower levels of endogenous SA exhibited shorter seedlings and reduced length of adventitious roots suggesting that SA can have varying impacts on different plant species or parts of plants with regards to growth (Xu et al. 2017). Likewise, the inhibitory or growth‐promoting effects of exogenous SA also vary, based on the concentration and species (Kováčik et al. 2009; Kazemi 2013; Pancheva et al. 1996; Pasternak et al. 2019; Bagautdinova et al. 2022). Hence, the threshold SA concentration that separates growth promotion from growth inhibition appears to be species‐specific (Li et al. 2022).

This balance that SA maintains between defence and growth is mainly mediated via crosstalk with other hormones, especially the growth‐promoting auxin hormone (Zhong et al. 2021). Auxin plays a key role in regulating plant growth especially root architecture. Auxin is known to control primary roots elongation and lateral root formation. The response of plants to auxin depends on the concentration and the type of tissue (Gomes and Scortecci 2021; Zhang et al. 2022). Studies have shown that during plant response to pathogen, increased SA production can repress auxin synthesis, auxin signalling and transport (Zhong et al. 2021; Yuan et al. 2017). However, it was recently shown that SA can promote auxin accumulation in roots. Exogenous treatment of Arabidopsis seedling with different concentrations of SA demonstrated that low concentrations enhanced adventitious roots and altered architecture of the root apical meristem by activating auxin synthesis, while high concentrations inhibited root growth. Based on these results it was suggested that there exist two bioactive concentration ranges for SA. At lower concentrations, it functions as a developmental regulator whereas at higher concentrations, it serves as a stress hormone (Pasternak et al. 2019). This supports the notion that SA‐mediated regulation of plant growth may operate through molecular mechanisms distinct from those involved in immunity. Biochemical studies have identified several potential SA‐binding proteins but their roles in SA signalling are still not well understood (Pokotylo et al. 2022).

In our study, LC‐MS analysis showed increased auxin levels when pak choy was exposed to either sweet basil or spearmint VOCs. Both hormones, SA and especially auxin, play crucial role in regulating plant growth. However, they influence growth in a concentration‐dependent manner in most tissues (Zhang et al. 2022; Li et al. 2022). Similar to SA, auxin levels were higher in plants exposed to sweet basil VOCs compared to those exposed to spearmint VOCs. It is plausible that sweet basil VOCs, unlike spearmint VOCs, can trigger the right bioactive concentrations of SA and auxin to initiate the downstream signalling required to promote shoot and root growth. This concentration‐dependent interplay between auxin and SA signalling pathways requires further investigation by multiplexing with recently developed nanosensors for real‐time, in vivo auxin monitoring (Khong et al. 2025).

Most research indicating that VOCs can influence auxin biosynthesis and transport to enhance plant growth is linked to microbial VOCs (Russo et al. 2022; Bailly et al. 2014; Fincheira and Quiroz 2018). For example, VOCs of soil‐borne fungus, *Fusarium oxysporum*, enhanced shoot and root growth of Arabidopsis and tobacco plants by regulating auxin transport and signalling (Bitas et al. 2015). VOCs emitted by a rhizobacteria *Bacillus* sp. JC03 increased biomass of Arabidopsis and tomato plants through auxin and strigolactone pathways (Jiang et al. 2019). Here, the increased root biomass observed in pak choy when exposed to sweet basil VOCs can possibly be attributed to the increased IAA content.

Research exploring the molecular mechanisms of VOC‐mediated promotion of plant growth under normal conditions is mainly derived from rhizosphere microbial VOCs studies. Using “*omics*” technology, studies indicate that microbial VOCs can alter photosynthesis, nutrient acquisition, hormonal and metabolic pathways to increase plant growth and stress response (Fincheira and Quiroz 2018; Fincheira et al. 2021; Rani et al. 2023; Poveda 2021). Our transcriptomics analysis showed that sweet basil VOCs can influence key primary metabolic pathways like carbon and nitrogen metabolism in pak choy, potentially altering overall plant performance. Plants depend on absorbed carbon and nitrogen for their optimal growth and development. Carbon and nitrogen metabolism are closely connected. Primary carbon metabolism relies on nitrogen assimilation since a significant portion of the plant's nitrogen resources is allocated to synthesize proteins and chlorophyll. Similarly, nitrogen metabolism and assimilation require energy, reducing power and carbon skeletons generated by carbon metabolism. Therefore, impacting one pathway greatly influences the other. A high level of CO_2_ assimilation promotes increased nitrogen assimilation and vice versa (Baslam et al. 2020; Aluko et al. 2023).

All photosynthetic life forms utilize the CBC cycle to convert atmospheric CO_2_ into organic materials necessary for growth and development. In recent years, a significant amount of research has centred on enhancing photosynthesis by overexpressing enzymes of the CBC cycle (Zhao et al. 2020; Khan et al. 2024). Exposure to sweet basil VOCs was able to increase expression of several enzymes in the CBC cycle of which overexpression of some are known to enhance photosynthesis and yield.

Ribulose bisphosphate carboxylase/oxygenase activase (RCA) is an enzyme that enables Rubisco function by changing its conformation (Waheeda et al. 2023; Sparrow‐Muñoz et al. 2023). Numerous research efforts have examined the beneficial link between higher RCA expression and plant photosynthesis and growth especially under elevated temperatures (Sparrow‐Muñoz et al. 2023; Qu et al. 2022). Interestingly, SA application in a dose‐dependent manner is shown to promote photosynthetic capacity of plants by regulating photosynthesis‐related genes including RCA, particularly under abiotic stress conditions (Shao et al. 2018; Dai et al. 2019). Exposure to spearmint VOCs was able to increase expression of RCA but at much lower levels, suggesting that appropriate level of SA induction is required to activate the enzyme levels to physiologically relevant concentrations.

Increased expression of chloroplast fructose‐bisphosphate aldolase is shown to improve plant growth and increase biomass production in tobacco and tomato (Uematsu et al. 2012; Cai et al. 2022). Transgenic tobacco plants that constitutively express phosphoenolpyruvate carboxylase (PEPC) from agave plants exhibited increased photosynthetic rates and biomass yield under normal conditions and improved drought and salt tolerance (Liu et al. 2021). We observed that genes encoding both these enzymes were upregulated in pak choy exposed to sweet basil VOCs.

Nitrogen fertilizer is the second most essential input required for crop production following water, and nitrogen is the most prevalent nutrient deficiency that limits yield. Mostly, plant roots uptake nitrogen in the form of nitrate or ammonium from the soil. Nitrate is reduced by nitrate reductase (NR) to nitrite, which gets imported into the chloroplast and further reduced to ammonium by nitrite reductase (NiR). Ammonium is then assimilated through the coordinated action of glutamine synthetase (GS) and glutamate synthase (GOGAT) to generate glutamine and glutamate, respectively, which act as nitrogen donors to produce other amino acids (Baslam et al. 2020; Zayed et al. 2023; The et al. 2021). Efforts have been made to alter the expression of these nitrogen metabolic genes to boost yield. For example, a recent study showed that in Rice, a higher activity of nitrate reductase enhanced grain yield and NUE (Gao et al. 2019). Constitutive expression of alfalfa NADH‐GOGAT in tobacco demonstrated increased biomass under sufficient nitrogen conditions (Chichkova et al. 2001). Higher kernel yields were observed in maize and wheat by overexpression of GS1 gene (Amiour et al. 2021; Habash et al. 2001). Sweet basil VOCs were able to increase expression of several enzymes such as nitrate reductase, nitrite reductase, and cytosolic CS isozyme 1‐2 (GS1‐2) that are involved in nitrogen assimilation in pak choy leaves by 2 h. SA application is shown to enhance the activity of NR, NiR, GS and GOGAT enzymes, thereby enhancing nitrogen metabolism, which aids to alleviate stress (Liang et al. 2023; Nazar et al. 2011; Hayat et al. 2014; Mahajan et al. 2023; Sangwan et al. 2015).

Overexpression of NR or NiR is also associated with reduced nitrate levels in leaves indicative of higher nitrogen assimilation (Davenport et al. 2015; Curtis et al. 1999). Estimation of nitrate leaves in pak choy leaves exposed to sweet basil VOCs demonstrated low levels of nitrate content. In addition to being a valuable source of essential nutrients, many green leafy vegetables, including Brassica species, tend to accumulate unsafe levels of nitrates, mainly due to excessive fertilizer application. Lower nitrate levels in vegetables can enhance the efficiency of nitrogen use in crops and reduce the health risks to humans associated with the conversion of nitrate to nitrite in vegetables (Luo et al. 2006; Bian et al. 2020). Improvement in root architecture such as length, surface area and density of root hairs promotes higher uptake of nitrogen in plants (Govindasamy et al. 2023). The changes in root architecture observed will also facilitate higher uptake of nitrogen. However, the uptake of extra nitrogen must align with the plants' metabolism to prevent systemic feedback regulation of metabolites that reflect the overall nitrogen status of the plant (Govindasamy et al. 2023). The transcriptome data suggest that very early upon VOC perception from sweet basil, pak choy plants concurrently modulate expression of key genes associated with improved nitrogen assimilation. Concurrent changes in root architecture and enhanced expression of nitrogen assimilation genes likely improve the NUE of the pak choy plants when grown with sweet basil, thereby positively influencing growth. The developmental stage at which root biomass begins to differ between monocropped pak choy and pak choy intercropped with sweet basil needs to be further examined.

Sulphur metabolism is tightly linked to nitrogen metabolism in plants. It is an important structural component of protein disulphide bonds, amino acids such as methionine and cysteine, vitamins, cofactors, and multiple specialized metabolites such as glucosinolates (Narayan et al. 2023; Takahashi et al. 2023).

Most *Brassicaceae* crops have higher sulphur requirements than other large crop species, such as wheat or maize as sulphur is required for glucosinolate production. Glucosinolate represent a category of secondary metabolites that are exclusive to plants belonging to the *Brassicaceae* family (Abdallah et al. 2010). SA is proposed to play a role in regulating sulphur metabolism. In *Brassica*, exogenous SA has been shown to increase the activity of sulphur assimilation enzymes, such as ATP sulfurylase, serine acetyl transferase, which are found to be upregulated by sweet basil VOCs in pak choy (Nazar et al. 2015). Overall, the enhanced carbon, nitrogen, and sulphur metabolism induced by sweet basil VOCs is likely the primary factor leading to the enhanced biomass of pak choy.

Apart from increased biomass, pak choy plants exposed to sweet basil VOCs also demonstrated higher accumulation of secondary metabolites, resulting in a significant enhancement in the nutritional quality of the crop. Plant secondary metabolites play an important role in plant stress response (Mocniak et al. 2023). SA is known to effectively promote the synthesis and accumulation of various secondary metabolites in plants (Ali 2021; Miao et al. 2024). Application of SA has been shown to improve glucosinolate content in several Brassica vegetables like Chinese cabbage, radish, and broccoli sprouts (Yi et al. 2016; Ghassemi‐Golezani et al. 2020; Kiddle et al. 1994). In harvested broccoli, SA treatment increased glucosinolates, thereby reducing the post‐harvest decline in quality (Yang, Luo, et al. 2023). SA treatments are also known to increase flavonoid and carotenoid accumulation in crops (Skrypnik et al. 2022; Gondor et al. 2016; Wang et al. 2024; Phong Lam et al. 2024). For example, in harvested oranges, SA treatment enhanced flavonoid and carotenoid accumulation (Huang et al. 2008). Foliar sprays of SA also resulted in higher amounts of flavonoid in cucumber fruits (Preciado‐Rangel et al. 2019). Moreover, application of SA has resulted in increased concentration of flavonoids and carotenoid in tomato (Gorni et al. 2021). The increased SA production observed in pak choy when exposed to sweet basil VOCs is a possible reason for the enhanced synthesis of these secondary metabolites.

Pak choy exposed to sweet basil VOCs displayed enhanced drought recovery and better tolerance to low nitrogen conditions. Drought negatively affects several metabolic processes including carbon, nitrogen and sulphur metabolism and secondary metabolic pathways (Feng et al. 2023; Marino et al. 2006). SA plays a crucial role in enhancing crop resilience to various abiotic stresses (Yang, Fang, et al. 2023; Altaf et al. 2023). There is more research on the effect of exogenous SA promoting in plant growth under stress conditions than under normal conditions. Numerous studies have demonstrated that application of SA is able to reduce the impact of stresses on plant growth, by regulating several important processes like photosynthesis, carbohydrate metabolism, nitrogen metabolism, proline metabolism, redox regulation, sulphur metabolism and regulation of water status (Yang, Fang, et al. 2023; Miura and Tada 2014; Ali et al. 2023; Majeed et al. 2024). This protective role of SA was also recently demonstrated in *Brassica napus* plants subjected to water and salinity stress (Islam et al. 2023; Iqbal et al. 2022; Ali et al. 2023). The augmented carbon, nitrogen, and sulphur metabolism along with increased secondary metabolites and improved root architecture observed in pak choy plants exposed to sweet basil VOCs, likely primes the plants and improves their stress resilience.

The above results also indicate the existence of an optimal bioactive concentration for SA that can promote stress resilience without compromising plant growth. In this study, we have analysed two abiotic stress conditions (drought and nitrogen stress) and further investigation is needed to demonstrate if exposure to sweet basil VOCs has primed pak choy to any other abiotic or biotic stress adaptations. Similarly, although no growth enhancement was observed in pak choy exposed to spearmint VOCs, it remains to be determined whether this exposure and the ensuing signalling has primed pak choy for other kinds of stress adaptation. For instance, a previous study has demonstrated that when *Brassica rapa* receiver plants were cultivated with peppermint plants, they displayed augmented anti‐herbivore ability (Sukegawa et al. 2018). Future work is needed to determine whether a brief exposure to VOCs is adequate to trigger the observed changes in pak choy or if sustained exposure is necessary. Transcriptome data indicate that changes happen early upon VOC perception and that the effects of VOCs are dosage dependent. It also remains to be examined whether the response of pak choy changes with increasing number of neighbouring aromatic plants, or if there is a specific range of VOC concentrations, above and below which pak choy plants does not respond.

Cells possess a finite set of signalling pathways that needs to translate information from various external stimuli into appropriate responses. The combined effects of the functions of signalling molecules involved, supported by their precise spatiotemporal control and concentration, play a key role in generating stimulus‐specific outcomes (Su et al. 2021; Peck and Mittler 2020). Our study shows that within 2 h, generation of ROS, SA and IAA is observed in pak choy exposed to sweet basil or spearmint VOCs, but it varies spatiotemporally and in quantity. This initial disparity in signalling upon VOC perception likely leads to the distinct transcriptional responses in pak choy, which in turn orchestrates the variations observed in growth and stress tolerance. Changes in other plant hormones and signalling molecules at the early stages of VOC perception need to be further investigated using multiple nanosensors.

VOCs can serve as a natural and environmentally friendly approach to improve both crop protection and yield. In our study, we demonstrate how VOC‐mediated signalling between companion plant sweet basil and receiver pak choy enhances the biomass of pak choy, and improves its water use and NUE. Additionally, the secondary metabolites content, which has important nutritional implications, is also enhanced. Choosing appropriate companion plants with different VOC emission profiles can be utilized in intercropping systems as a sustainable strategy of harnessing VOC‐driven signalling to enhance crop productivity and stress resilience. Alternatively, plants can also be exposed to synthetic beneficial VOCs instead of companion plants, especially in confined environments like indoor farms and greenhouses. However, before the VOC‐mediated communication among plants can be effectively harnessed for sustainable agriculture, it is imperative to decipher the plant VOC sensing code. The mechanisms by which plants perceive and respond to VOCs are just beginning to be comprehended. The development of microneedle arrays embedded with different nanosensors can enable simultaneous, multiplexed detection of multiple biochemical molecules involved in VOC‐triggered signalling. This technology offers a unique opportunity to decode the signal transduction mechanisms underlying VOC‐mediated communication. Such studies would deepen our understanding of VOC‐mediated communication in plants and its ecological significance.

## 4 Materials and Methods

### 4.1 Preparation of H_2_O_2_ and Reference Sensors Based on DNA‐Wrapped SWNTs

H_2_O_2_ and reference sensors are SWNTs wrapped with single‐stranded DNA oligomers (GT)_15_ and (AT)_15_, respectively, that were purchased from Integrated DNA Technologies. Purified HiPco SWNTs were purchased from NanoIntegris (Lot #HP32‐0180) while CoMoCAT (6,5)‐chirality enriched SWNTs were purchased from Sigma Aldrich. To generate (GT)_15_‐SWNT, 1 mg of single‐stranded (GT)_15_ was mixed with 0.25 mg of HiPCO SWNT in 1 mL of 100 mM NaCl. To generate (AT)_15_‐SWNT, 1 mg of single‐stranded (AT)_15_ was mixed with 0.25 mg of CoMoCAT (6,5)‐chirality SWNT in 1 mL of 100 mM NaCl. The mixtures were sonicated with a 3‐mm probe tip (Qsonica Q500) for 15 min at 22% amplitude in an ice bath. The samples were then centrifuged twice at 16,000*g* for 90 min each at 20°C to remove remaining SWNT aggregates.

### 4.2 Synthesis of Co‐Polymer Wrapping of SA Sensor

The SA sensor is made up of purified HiPco SWNT wrapped with a cationic fluorene‐pyrazine (PFPz) co‐polymer synthesized in‐house. To synthesize the co‐polymer, reagents, monomers, catalysts, and solvents were purchased from Sigma‐Aldrich Ltd. and Tokyo Chemical Industry Ltd., unless otherwise stated. Prior to polymerization, dimethylpropylamine side chains were attached to the 9‐H position of the 2,7‐dibromofluorene monomer (F‐diBr) as precursors to cationic functionalization. To enable the Suzuki cross‐coupling process, the di‐bromo (diBr) groups of the fluorene monomer were then converted to di‐boronic ester groups (diEs). The PFPz co‐polymer was synthesized via microwave‐assisted Suzuki polycondensation, a Pd‐catalysed polymerization technique. In the presence of 3 mol% catalyst 1,1′‐bis(diphenylphosphino)ferrocene] dichloropalladium(II) (Pd(dppf)Cl_2_), equimolar quantities of F‐diEs and 2,6‐dibromo pyrazine (Pz‐diBr) were mixed together with stirring in a 20‐mL microwave‐safe vial. The vial was crimp sealed, pumped down to vacuum and backfilled with Ar thrice. Degassed anhydrous THF:DMF (2:1) were added to the vial to dissolve the monomers, followed by degassed Na_2_CO_3_ solution (5 eq). The resultant reaction mixture was heated in a microwave reaction chamber (Biotage Intiator+ Microwave Synthesizer) at 130°C for 15 min. Upon cooling to room temperature, the mixture was precipitated in water and obtained by filtering through a 0.45‐μm nylon filter. The polymer was then washed with water until the washing was neutral (pH = 7) to remove all Na_2_CO_3_. The polymer solids were re‐dissolved in THF and filtered again through a 0.45‐μm nylon syringe filter to remove other insoluble impurities. To the filtered polymer solution, sodium diethyldithiocarbamate solution in methanol (100 mg mL^−1^) was added (100 eq) and stirred for 3 h to remove Pd catalyst residues. The purified PFPz co‐polymer was then retrieved again by re‐precipitation and repeated washing in water to remove excess sodium diethyldithiocarbamate. To allow cationic functionalization, the PFPz co‐polymer (100 mg) was dissolved in THF (4 mL) in a 20‐mL vial, followed by the addition of 1 M HCl (4 mL). Upon addition of HCl, the polymer solution turned cloudy and re‐dissolved when deionized water was added (12 mL). The solution was then transferred to a cellulose ester dialysis tube (3–5 kDa MWCO) for dialysis with deionized water for 2 days. The external dialysate of deionized water was refreshed every 8 h to aid removal of excess HCl, THF and oligomers. The purified cationic PFPz co‐polymer was finally retrieved via freeze‐drying.

### 4.3 Preparation of SA Sensor Based on Cationic PFPz‐Wrapped SWNT

To generate PFPz‐wrapped SWNT, 2.5 mg of cationic PFPz co‐polymer was dissolved in 1 mL deionized water, before the addition of 1 mg of HiPco SWNT powder. The mixture was sonicated with a 3‐mm probe tip (Qsonica Q500) for 30 min at 25% amplitude in an ice bath. To remove the remaining SWNT bundles, the PFPz‐wrapped SWNT was ultra‐centrifuged at 153,145*g* for 2 h.

### 4.4 In Vitro Optical Characterization of Nanosensors

UV–Vis–NIR spectrophotometry (Agilent Cary 5000) was used to determine SWNT concentration by measuring SWNT absorbance at 632 nm with an extinction coefficient of 0.036 (mg/L)^−1^ cm^−1^ and to confirm the presence of SWNT E11 bands of various SWNT chiralities. Typically, (GT)_15_‐SWNT concentrations are 30–50 mg L^−1^, (AT)_15_‐SWNT concentrations are 120–150 mg L^−1^ and PFPz‐SWNT concentrations are 60–80 mg L^−1^. Photoluminescence excitation (PLE) measurements, performed on a Jobin‐Yvon Nanolog‐3 spectrofluorometer coupled with an InGaAs detector, were used to measure the SWNT fluorescence before and after the addition of H_2_O_2_ and SA analytes and to ensure the repeatability of sensor performance over different SWNT batches generated. SWNT samples were excited at 785 nm with a bandwidth of ±25 nm, while nIR emission was collected in the wavelength range of 900–1600 nm, with an integration time per emission scan set at 30 s. For each SWNT batch generated, we confirmed that (GT)_15_‐SWNT gives a selective quenching response to H_2_O_2_ and PFPz‐SWNT gives a selective quenching response to SA, whereas (AT)_15_‐SWNT remains invariant to both analytes. Magnitude for H_2_O_2_ and SA sensor responses was measured for each sensor batch generated to ensure consistency with reported calibration curves (Ang et al. 2024; Lew et al. 2020).

### 4.5 Leaf Infiltration With Nanosensors

H_2_O_2_ sensor (5 mg L^−1^) diluted in deionized water was infiltrated into the abaxial side of the selected leaf in pak choy using a 1 mL needleless syringe. Prior to infiltration of the SA sensor, free PFPz polymer that is unbound to SWNT was removed by repeated centrifugal filtration (Amicon Ultra‐4 Centrifugal Filter Tubes, 100 kDa MWCO). Complete removal of free PFPz polymer was ascertained by the absence of PFPz polymer UV–Vis absorbance band (*λ*_max_ = 370 nm) in the filtrate. Upon removal of free polymer, SA sensor (2.5 mg L^−1^) was infiltrated in the same pak choy leaf, partitioned by a dominant pak choy leaf vein to minimize migration and mixing of sensors. Across the leaf midvein, reference sensor (1 mg L^−1^) was infiltrated. For all sensor infiltrations, gentle pressure was applied on the other side of the leaf to minimize tissue damage during infiltration.

### 4.6 Detection of H_2_O_2_ and SA Signals Upon Intercropping Using Multiplexed Nanosensors

Pak choy was used as a receiver plant and separately exposed to 2 different emitter plants (sweet basil and spearmint) grown in isolated pots, as well as disks containing individual VOCs (eugenol and carvone). Perspex boxes (30 × 30 × 30 cm) were used to contain the receiver plants, and emitter plants or VOC disks. Receiver pak choy and emitter sweet basil/spearmint plants were arranged in the well‐aerated box in a ratio of 1:2. Prior to measurement, the receiver pak choy plant was infiltrated with H_2_O_2_, SA and reference sensors on the same leaf in distinct areas of the leaf and equilibrated for 1 h. Upon equilibration, the pak choy plant was brought into the perspex box and placed in front of the NIR imaging standoff system, comprising a liquid‐N_2_‐cooled Princeton Instruments NIRvana InGaAs detector coupled to a Nikon AF‐S Micro‐NIKKOR 105‐mm f/2.8D lens. The sensor‐infiltrated pak choy plant was then secured with tape at a distance of approximately 1 m from the camera lens, with an 830‐nm long‐pass filter (Semrock Razor Edge LP02‐830) placed in front of the camera lens. One side of the perspex box was kept open to facilitate uninterrupted passage of laser (Incident power: 32 mW cm^−2^) and capture of SWNT sensor NIR fluorescence (Wavelength: 900–1700 nm) images by the near‐infrared imaging standoff system over time. The laser excitation spot on the leaf is circular with radius of approximately 13.5 mm, covering the multiple sensor infiltration sites on the leaf. After 15 min of measurement to ensure baseline stability, 2 aromatic plants (sweet basil/spearmint) were kept next to the infiltrated pak choy to mimic intercropping conditions. As control, 2 pak choy plants were kept next to the infiltrated pak choy plant to mimic monocropping conditions. Changes in the respective fluorescence of each sensor were further monitored for 2 h. For pure VOC exposure, instead of aromatic plant, 80 µL of either 100% eugenol or carvone was added to a 1.5 mL Eppendorf tube cap and placed near the pak choy receiver plant.

### 4.7 Application of Diphenyleneiodonium (DPI) ROS Inhibitor

DPI was purchased from Sigma Aldrich and dissolved at 2.5 mM in DMSO solvent before further dilution to 100 μM in sterile water. Pak choy leaf was first infiltrated with DPI solution 1 h prior to SA, H_2_O_2_ and reference sensor infiltration on distinct areas of the same leaf. Upon 1 h of equilibration, fluorescence measurements commenced inside the Perspex box using the abovementioned optical conditions. As control, blank solution (1:25, DMSO: sterile water) was infiltrated into the leaf instead of DPI. Following 15 min of fluorescence measurement to ensure baseline stability, 2 sweet basil or spearmint plants were brought into the Perspex box next to the sensor‐ and DPI‐infiltrated pak choy plant. Changes in sensor fluorescence were then examined for 2 h.

### 4.8 Image Analysis

Image and data analysis were performed using ImageJ and Matlab R2024a. For analysis of each sensor, a custom Matlab R2024a script was used to select the distinct sensor regions of interest by applying appropriate image contrast and thresholds. After capturing the sensor fluorescence intensities over time, time plots of sensor responses to intercropping conditions are normalized to the initial values, which represent the basal SA/H_2_O_2_ levels present in the leaf prior to respective intercropping conditions at *t* = 15 min. We then focus on detecting the normalized fluorescence intensity deviation post intercropping. To obtain a ratiometric sensor response, the normalized intensities are divided by the reference sensor normalized intensity to account for leaf movement and laser intensity drifts that could potentially convolute the results. The ratiometric data were then smoothed with a moving average filter using a window width of 10 time points. Concentration maps for SA/H_2_O_2_ detected within the imaged leaf areas could then be derived from the fluorescence intensity changes using the respective in vitro sensor calibration curves (Ang et al. 2024; Lew et al. 2020). Detailed image processing and analysis steps using Matlab R2024a are described in Supplementary Information, Supplementary Figure 7.

### 4.9 Plant Growth and Conditions

Pak choy plants were grown in a controlled environment chamber under long days (16 h) at 25°C and 70% humidity. Aromatic plants, sweet basil and spearmint were grown in separate controlled environment chambers under same conditions and were used for intercropping set‐up.

### 4.10 Intercropping Experiment Design

Six‐millimetre‐thick clear Perspex chambers (*L* 500 mm × *W* 200 mm × *H* 400 mm) were custom made for our intercropping box experiments. Perspex chambers were placed in controlled environment rooms under long days (16 h) at 25°C and 70% humidity. Pak choy seeds of commercial variety were obtained from Ban Lee Huat Seeds, Singapore. Seeds were germinated and 5‐day‐old pak choy seedlings were transferred to small pots containing equal amount of commercial growing medium made from organic peat moss, and were placed in the chamber with one basil plant on both sides. The chambers were not fully sealed with the doors left slightly ajar to prevent accumulation of moisture caused by plant transpiration and condensation. For monocropping setup, three pak choy plants were placed in a similar Perspex box. All plants were watered similarly and grown for 5 weeks. We further repeated the experiment in standard plant growth chambers (A1000 Plant Growth Chamber) with a ratio of 3 pak choy: 1 sweet basil. One growth chamber was used for monocrop and another chamber of same size and setting was used for intercropping experiments.

### 4.11 Low Nitrate Tolerance Study

Vermiculite was used as nutrient‐free soil for evaluating the growth of pak choy under low nitrate conditions. It was washed three times with deionized water to remove any soluble nutrient and air‐dried at room temperature. Vermiculite was then irrigated with Hoagland's solution containing 2.5, 1.5, 1, 0.5, and 0 mM nitrate. Five‐day‐old seedlings of pak choy were transferred to vermiculite and grown at the above‐mentioned nitrate concentrations. Plants were irrigated twice a week with equal volume of respective nutrient solutions. At 5 weeks of growth, parameters, such as biomass and root weight, were analysed. Intercropping experiments were conducted in growth chambers (A1000 Plant Growth Chamber) at a ratio of 3 pak choy: 1 sweet basil under 1.5 mM nitrate condition, with monocropped pak choy used as control.

### 4.12 Drought Resistance Test and Recovery

Five‐day‐old pak choy seedlings were transferred to 18‐well trays having equal amount of potting medium in each well, and trays were separated into two groups. One group was intercropped with sweet basil at a ratio of 3 pak choy: 1 sweet basil, and control group was monocropped with pak choy in two different growth chambers under same conditions. Plants were watered twice weekly with equal amount of water. When the plants were 3 weeks old, lower trays were filled with water to allow the soil in the trays to become fully saturated, and subsequently, water from lower tray was drained fully. Saturating of the soil before the initiation of the drought stress helps to minimize any variation in soil moisture between individual wells. After this, watering was withheld for 16 days and Leaf RWC was measured on Days 0, 10 and 16th day of the trial using the formula $\mathrm{RWC}=\frac{\mathrm{FW}-\mathrm{DW}}{\mathrm{TW}-\mathrm{DW}}\times 100$, where FW is the fresh weight, TW the turgid weight and DW the dry weight. On Day 16, both trays were watered again to saturation. Recovery was measured 72 h post re‐watering.

### 4.13 Measurement of Plant Hormone Levels by LC‐MS

For hormone measurements, 700 mg of pak choy leaves from sweet basil/spearmint intercropped for 30 min, 1 h and 2 h and monocropped control were harvested into a 2 mL microfuge tube. 1 mL of 80% methanol was added to each tube and ground in a bead beater (Qiagen) with 3 mm tungsten beads at 25 Hz s^−1^ for 3 min. Samples were placed on ice for 30 min followed by centrifuging at 13,000*g* for 10 min at 4°C. Supernatant was transferred to a new 2 mL microfuge tube. The extract was dried under the flow of nitrogen and resuspended in 200 µL of 100% Methanol and filtered using 0.22 µm centrifuge tube filter (Corning, USA) and used for hormone measurements. The filtered plant extract (5 µL) was injected into UPLC–MS/MS system for hormonal analysis. The analysis was conducted using an UPLC system coupled to a triple quadrupole spectrometer (Agilent 6490 triple quadrupole MS equipped with Jet stream electrospray ionization source (Agilent Technologies, Santa Clara, USA). Phytohormones were separated on a Phenomenex C18 reversed phase column (2.1 mm × 100 mm, 1.7 µm) maintained at 45°C. The mobile phase (A) was composed of 0.1% formic acid in water and 100% methanol constituted mobile phase (B). The elution gradient started with 5% methanol for 1–5 min, then increased linearly to 100% at 10–18 min, followed by decrease to 5% at 20 min. Oven temperature was maintained at 40°C. Detection was performed in MRM monitoring mode. The MS parameters included drying gas temperature, 350°C; gas flow rate, 12 L min^−1^; nebuliser pressure, 35 psi; sheath gas temperature, 400°C; sheath gas flow rate, 12 L min^−1^; delta electron multiplier volt, 500 V; and capillary voltage, 4000 V in the negative ionization mode. The data were analysed using Quantitative Analysis Mass Hunter Workstation software for QQQ (Agilent Technologies, Santa Clara, CA, USA). A calibration curve with hormone standard was used for quantification. Analytes were then quantified based on the standard curve to determine the concentrations.

### 4.14 Estimation of Total Flavonoid Content (TFC)

Aluminium trichloride method was used for the determination of flavonoids content as described by Zhishen et al. (1999), using quercetin as reference compound. Methanolic extract was prepared by homogenizing 0.1 g of leaf from pakchoi plants intercropped with sweet basil for 5 weeks as well as monocropped control in a 2 mL vial with 80% methanol. A volume of 500 μL of leaf extract was added to 150 μL of sodium nitrite solution (5%), vortexed and allowed to stand for 5 min. The mixture was then incubated for 6 min after the addition of 150 μL of aluminium trichloride (10%), followed by the addition of 2 mL of sodium hydroxide (5%). The final volume of the solution was adjusted to 5 mL with distilled water. After 15 min of incubation, the absorbance was measured by using a plate reader (Tecan Spark Microplate Reader, Switzerland) at 510 nm. The total flavonoids content was expressed as milligrams of quercetin equivalents per gram of plant extract (mg g^−1^).

### 4.15 Estimation of Total Carotenoid Content (TC)

The total carotenoid content in the leaves was analysed by the modified method of Kaisangsri et al. (2016), using β‐carotene as the reference compound. 80 mg of leaf sample and 4 mL of dimethyl sulfoxide (DMSO) was added to the glass tube, incubated at 75°C in a water bath for 50 min, and cooled to room temperature. After that 4 mL n‐hexane, containing 0.1 g/100 mL butylated hydroxytoluene was added, vortexed for 10 s, and left for 30 min. Two to three drops of ethanol was added to precipitate any protein in the hexane phase. The top layer was removed into another tube using a glass pipette. Approximately 0.5 g of anhydrous sodium sulphate was added to the tube and let stand for 15 min to absorb any residual water in the n‐hexane solution. Total carotenoid concentration was measured using the absorbance at 450 nm using a plate reader (Tecan Spark Microplate Reader, Switzerland). To determine total carotenoid concentration, a standard curve was created using known concentrations of β‐carotene (0–1 mg L^−1^) dissolved in hexane.

### 4.16 Estimation of Total Glucosinolate Content

The spectrophotometric estimation was performed using a methanolic extract prepared from 0.1 g of leaf tissue from pak choy plants, which were intercropped with sweet basil for 5 weeks, as well as from a monocropped control. The extraction was carried out in a 2 mL vial using 80% methanol, following the modified method of Mawlong et al. 2017 (Mawlong et al. 2017). Glucobrassicin was used as a reference compound. This homogenate was kept overnight at room temperature and centrifuged at 3000 rpm for 4 min. The supernatant was collected and made up to 2 mL with 80% methanol. 100 µL of this extract was used for glucosinolate estimation. To this, 0.3 mL of double‐distilled water and 3 mL of 2 mM sodium tetrachloropalladate solution (prepared by dissolving 58.8 mg of sodium tetrachloropalladate in 170 µL of concentrated HCl and diluting to 100 mL with double‐distilled water) were added. After incubation at room temperature for 1 h, absorbance was measured at 425 nm using a plate reader (Tecan Spark Microplate Reader, Switzerland). The total glucosinolate content was expressed as milligrams of Glucobrassicin equivalents per gram of plant extract (mg g^−1^).

### 4.17 RNA Sequencing (RNA Seq)

Pak choy leaves from sweet basil/spearmint intercropped for 2 h or monocropped control were collected and ground to fine powder using liquid N_2_. Total RNA was extracted using RNeasy Plant Mini kit from Qiagen following the manufacturer's protocol (Cat. No. 74904). The quality of RNA was checked by measuring the ratio of OD260 to OD280 and the integrity was evaluated by measuring the RNA Integrity Number (RIN) number using Agilent 2100 bioanalyser. Sequencing, assembly and annotation services were provided by Novogene AIT Genomics Singapore. Briefly, messenger RNA was purified from total RNA using poly‐T oligo‐attached magnetic beads. After fragmentation, the first strand cDNA was synthesized using random hexamer primers, followed by the second strand cDNA synthesis using either dUTP for directional library or dTTP. Quantified libraries were pooled and sequenced on Illumina platform, according to effective library concentration and data amount. Raw data (raw reads) of FASTQ format were first processed through fastp. Brapa_1.0 reference genome and gene model annotation files were downloaded from genome website browser (NCBI/UCSC/Ensembl) directly. Paired‐end clean reads were mapped to the reference genome using HISAT2 software. Differential expression analysis between two conditions/groups (three biological replicates per condition) was performed using DESeq. 2R package. The *p* values were adjusted using the Benjamini and Hochberg methods. Differential expression analysis between two conditions/groups (three biological replicates per condition) was performed using DESeq. 2R package. The resulting *p* values were adjusted using the Benjamini and Hochberg's approach for controlling the False Discovery Rate (FDR). Genes with an adjusted *p* value < 0.05 found by DESeq. 2 were assigned as differentially expressed. Gene Ontology (GO) enrichment analysis of differentially expressed genes was implemented by the cluster Profiler R package. Cluster Profiler R package was used to test the statistical enrichment of differential expression genes in KEGG pathways. The data have been deposited with links to BioProject accession number PRJNA1232253 in the NCBI BioProject database (https://www.ncbi.nlm.nih.gov/bioproject/).

### 4.18 Validation of RNA Seq by RT‐qPCR

Pak choy leaves from sweet basil/spearmint intercropped for 2 h or monocropped control were used for total RNA extraction using RNeasy Plant Mini kit from Qiagen following the manufacturer's protocol (Cat. No. 74904). The isolated RNA was converted into cDNA using iScript Reverse Transcription Supermix (BioBio‐RadRad) as per the manufacturer's protocol. To validate the DEGs, RT‐qPCR was performed using PowerUp SYBR Green Master Mix (Applied biosystems) at optimized conditions. A final volume of 10 µL of the reaction mixture was prepared using 10 ng of cDNA and 5 µL of the SYBR Green Master Mix and 100 nM of each gene‐specific reverse and forward primer. The final composition of the reaction mixture was kept same for DEGs and the reference gene. *Actin* was used as the internal reference. The fold change in expression of the selected DEGs was estimated from sweet basil/spearmint intercropped for 2 h and monocropped control. The comparative threshold (*C_t_*) cycle method (2–ΔΔ*C_t_*) was used for relative quantification and fold change calculations. The primer sequences are listed in Supplementary Table 4.

## Conflicts of Interest

The authors declare no conflicts of interest.

## Supporting information


Supporting File 1

Supporting File 2

Supporting File 3
