# Supplementary material for: Multiplexed Nanosensors for Real‐Time Monitoring of VOC‐Mediated Signalling Response in Plants
Source: Plant Cell Environ. 2026 Sep 2;49(12):9907–31. doi: 10.1111/pce.70844 (PMC13725256; doi:10.1111/pce.70844)
Supplement: Supplementary file 1 [file PCE-49-9907-s003.docx]

**Supporting Information**

**Multiplexed Nanosensors for Real-Time Monitoring of VOC-mediated Signaling Response in Plants**

Mervin Chun-Yi Ang^1,2#^, Jolly Madathiparambil Saju^3#^, Sayyid Mohaideen^1^, Niha Dhar^3^, Sarangapani Sreelatha^3^, Sayuj Koyyappurath^3^, Thomas K Porter^4^, Gabriel Sánchez-Velázquez^4^, Vaishnavi Reddy Amarr^3^, Song Wang^1,5^, Duc Thinh Khong^1^, Suh In Loh^1^, Michael S. Strano^1,4*,^ Sarojam Rajani^1,3*^

^1^Disruptive & Sustainable Technologies for Agricultural Precision IRG, Singapore-MIT Alliance for Research and Technology, 1 CREATE Way, #03-06/07/08 Research Wing, Singapore 138602, Singapore

^2^Natural Sciences and Science Education, National Institute of Education, Nanyang Technological University, 1 Nanyang Walk, Singapore 637616, Singapore

^3^Temasek Life Sciences Laboratory Limited, 1 Research Link National University of Singapore, Singapore 117604, Singapore

^4^Department of Chemical Engineering, Massachusetts Institute of Technology, 77 Massachusetts Avenue, Cambridge, MA 02139, USA

^5^State Key Laboratory of Mesoscience and Engineering, Institute of Process Engineering, Chinese Academy of Sciences, 1 North 2nd Street, Zhongguancun, Haidian District, Beijing 100190, China

*Corresponding authors’ email address: [rajanis@tll.org.sg](mailto:rajanis@tll.org.sg), and [strano@mit.edu](mailto:strano@mit.edu)

^#^ These authors contributed equally to this work.

**Supplementary Discussion**

**Modeling the Spatial Dynamics of VOC Transfer Between Plants**

To model the diffusion of VOCs emitted by sweet basil or spearmint and their subsequent uptake by a receiver plant (pak choy), we used a one-dimensional diffusion model:

$$\frac{dC}{dt}=D_{VOC}\frac{d^{2}C}{dx^{2}}$$

This equation governs the VOC concentration $C\left( x,t \right)$ as it evolves over time and space, where $D_{VOC}$ is the diffusivity of the VOC in air.

The initial condition assumes no VOC is present in the domain at time t=0:

$$C\left( x,t=0 \right)=0$$

The far-field boundary condition assumes an infinite domain, where VOC concentration approaches zero:

$$C\left( x\to\infty,t \right)=0$$

At the emitter plant interface (x=0), VOC flux into the domain is modeled using a mixed boundary condition that accounts for the compound’s saturation vapor pressure and the total mass transfer resistance from the leaf (contributions from both cuticle and stomata).

$$D_{VOC}\left. \frac{dC}{dx} \right|_{x=0}= {-k}_{m,e}\left( \frac{P_{sat}}{RT}-C\left( x=0,t \right) \right)$$

$$k_{m,e}=k_{cuticle}+k_{stomata}$$

Here:

- $k_{m,e}$ is the total mass transfer coefficient for the emitter plant
- $k_{cuticle} and k_{stomata}$ are the individual mass transfer coefficients for the cuticle and stomata
- $P_{sat}$ is the saturation vapor pressure of the VOC
- R is the ideal gas constant
- T is the temperature

In this formulation, VOC diffuses through the cuticle and stomata (assumed to act as parallel resistances) and are released into the surrounding air at a rate proportional to the difference between the internal concentration ($\frac{P_{sat}}{RT}$) and the VOC concentration just outside the leaf surface ($C\left( x=0,t \right)$). The use of $P_{sat}$ allows incorporation of VOC-specific properties and temperature dependence. We estimated $P_{sat}$ for each VOC using both the Antoine equation and Clausius-Clapeyron relation, drawing parameters from NIST-reported values^1, 2^. For modeling purposes, we assumed carvone as the representative VOC for spearmint and eugenol for sweet basil.

At the receiver plant interface, VOC uptake into the leaf is modeled as a first-order mass transfer process from the external concentration field into internal compartments within the plant. This process is described by the following ordinary differential equation:

$$V_{Receiver}\frac{dC_{Receiver}}{dt}=A k_{m,r}(C\left( x=d,t \right)-C_{Receiver})$$

where:

- $C\left( x=d,t \right)$ is the VOC concentration at the receiver plant surface (i.e., at distance x = d from the emitter)
- $C_{Receiver}$ is the VOC concentration in the internal compartment of the receiver plant
- $k_{m,r}$ is the total mass transfer coefficient for the receiver plant
- A is the effective surface area of the receiver tissue exposed to the VOCs,
- $V_{Receiver}$ is the volume of the compartments into which the VOC can accumulate

This formulation assumes that the rate of VOC accumulation inside the plant is driven by the concentration gradient between the external VOC level at the receiver surface and the internal VOC concentration. Once $C_{Receiver}$ exceeds a threshold value, downstream signaling (e.g. ROS or SA production) is triggered.

The gas-phase diffusivity ($D_{VOC})$ was fixed at 1 × 10^−6^ m^2^ s^-1^ (typical for organic vapours in air)^3^. Stomatal conductances reported for pak choy, spearmint, and sweet basil are in the 0.1, 0.1, and 0.01 mol H_2_O m^-2^ s^-1^, order of magnitude respectively^4-6^. For cuticular conductance, a value of 89 × 10^-6^ m s^-1^ was used for pak choy based on reported values for a similar Brassicaceae species (kai lan)^7^. For the emitter species, a cuticular conductance of 1 × 10^-6^ m s^-1^ was assumed, consistent with reported ranges for various plant cuticles^8^. Since these conductance values are reported for water vapor, we converted them to VOC conductance using the following scaling:

$$k_{VOC} =k_{H2O}(\frac{D_{VOC}}{D_{H2O}})$$

where $D_{H2O}$ and $D_{VOC}$ correspond to the diffusivity of the VOC and water in air respectively. To convert the stomatal conductance (mol m^-2^ s^-1^) to a mass transfer coefficient (m s^-1^), we divide them by P/RT (assuming ideal gas, and T and P at 25^o^C and 1 atm respectively). Finally, the A:V_Receiver_ ratio was set to 100 cm^2^ cm^-3^, consistent for edible leaves^9^.

VOC emission and perception have been shown to be both temperature and distance-dependent. Generally, higher temperatures can promote greater VOC emission, with the spectrum of volatiles emitted also showing a degree of temperature dependence^10, 11^. Furthermore, logically, the interplant distance has also been shown to play a role in VOC perception, with perception and VOC effects diminishing as the distance between plants increases^12, 13^. This modeling framework, the inclusion of compound-specific vapor pressure terms and their temperature dependence, along with diffusion governing equations capturing the distance dependence (i.e., lower VOC concentrations at longer distances), allows us to capture these two phenomena. Lastly, by explicitly incorporating both stomatal and cuticular conductances, the model also provides flexibility to simulate physiological variability; stomatal conductance is known to respond to light, temperature, and humidity^14, 15^. While we assumed constant values based on literature for standard growth conditions, this structure allows future modeling of dynamic responses under changing environmental stimuli.

**Detailed Image Processing and Analysis Steps for Nanosensors using Matlab**

For analysis of the real-time nanosensor fluorescence response to changes in H_2_O_2_ and SA in living plants, a custom Matlab R2024a script was used (Supplementary Figure 7), which automates (1) Image pre-processing, (2) Threshold adjustment and region of interest (ROI) detection, (3) Fluorescence measurement and normalization, (4) Data Visualization.

1. ***Image Loading and Pre-processing***

The pipeline begins by loading a multi-frame TIFF image using the MATLAB *uigetfile* function, allowing interactive file selection. The selected file is read frame by frame using the *imread* function, and the image data are stored in a 4D array (rawFrames). The script then checks the intensity level of the first frame, analyzing features like entropy and uniformity, to determine if contrast enhancement is necessary. If the intensity is low, making segmentation challenging, the image undergoes Contrast Limited Adaptive Histogram Equalization (CLAHE) using MATLAB's *adapthisteq* function, enhancing contrast in sensor regions. Otherwise, basic contrast stretching is applied. Gaussian noise is reduced using *imgaussfilt*, which applies a Gaussian filter with a specified standard deviation.

2. ***Threshold Adjustment and ROI Detection***

A graphical user interface (GUI) allows interactive threshold adjustment via a slider. The threshold determines the binary segmentation of the image, facilitating ROI identification. Once the optimal threshold is set, the "Start Measurement" button saves the threshold value and initiates the detection of connected components in the binary image. Small, insignificant components or noise are filtered out using the *bwareaopen* function, which removes objects below a specified pixel area. The pipeline then prompts the user to enter the integration time and the frame number at which emitter plants (e.g. sweet basil/spearmint) or a VOC (eugenol/carvone) disk is introduced. To ensure consistency, integration time is kept to 30 s per frame and emitter plants or VOC disks are introduced at the 15 min time-point (30th frame).

3. ***Measurement and Normalization***

For each detected ROI for SA, reference and H_2_O_2_ sensors, the pipeline computes the mean intensity across all frames. Temporal variations in mean intensity are plotted alongside the ROI boundary on the first frame, displayed in the jet colormap for enhanced visualization. The user is prompted to enter a name for each ROI, facilitating organized data storage. Mean intensity values are normalized to 1 by dividing them by the mean intensity of the first 30 frames for all sensor regions, which indicates the baseline levels of SA and H_2_O_2_ in the leaf prior to introduction of emitter plants or VOC disks. The data is further normalized against a reference sensor ROI to account for external variations in imaging conditions.

4. ***Data Visualization and Export***

The pipeline computes a moving average of the normalized intensity values for each ROI, smoothing the data to reduce noise. These smoothed datasets are plotted against time, with the time axis converted to hours for easy interpretation. Finally, processed data, including region names, mean values, and normalized ratios, are organized into a table and displayed in the MATLAB workspace. The table is saved for further analysis, and all normalized data are plotted, with the reference ROI highlighted for comparison.

**SUPPLEMENTARY FIGURES**

**Supplementary Figure 1** Modeling of VOC-mediated plant-to-plant signaling using a 1D diffusion framework. (a) VOCs emitted by an aromatic emitter plant diffuse through air toward a receiver plant across a distance *d*.(b) Left boundary condition: the flux of VOCs into the system is proportional to the difference between the VOC saturation concentration inside the emitter plant ($\frac{P_{sat}}{RT}$) and the VOC concentration in the air immediately outside the leaf surface ($C\left( x=0,t \right)$), modulated by a mass transfer coefficient that accounts for contributions from the cuticle and stomata ($k_{m,r}$). **(c)** Right boundary condition: upon reaching the receiver plant surface, VOCs diffuse through the leaf and accumulate in an internal compartment. This uptake is modeled as an ODE dependent on the local VOC concentration at the receiver surface $C\left( x=d,t \right)$, which triggers downstream signaling once a threshold is reached.

**Supplementary Figure 2** (a) Photograph illustrating a pak choy plant infiltrated with SA (green), H_2_O_2_ (red) and Reference (blue) sensors, which is exposed to a VOC disk containing either eugenol or carvone; Time-plots of H_2_O_2_ (red) and SA (green) concentration derived from nanosensor experiments whereby pak choy was exposed to a disk containing (b) eugenol and (c) carvone at t = 15 min (black arrow). Shaded regions represent standard error across 3 independent replicates.

**Supplementary Figure 3** Volcano plot showing (a) a total of 5358 differentially expressed (DE) genes of which 3538 were up-regulated (red) and 2300 were down-regulated (green) when pak choy plants were exposed to sweet basil; and (b) a total of 5931 DE genes of which 3001 were up-regulated (red) and 2930 were down-regulated (green) when pak choy plants were exposed to spearmint Genes that are not differentially expressed are represented by blue dots.

**Supplementary Figure 4** Average concentrations of IAA in pak choy as determined by LC-MS, at t = 0.5 h, 1 h and 2 h following exposure to either spearmint (green) or sweet basil (blue) companion plants. Pak choy grown with other pak choy plants served as the monocropped control (red). Bar graphs show the mean values with error bars representing standard deviations from independent experiments (*n* = 3). Individual data points are shown as dots. Different letters show significant differences using one-way ANOVA with a Tukey’s HSD test at *p* < 0.05.

**Supplementary Figure 5** (a) Biomass comparison of monocropped pak choy and pak choy intercropped with sweet basil (n = 36) that were grown for 5 weeks in a small growth chamber in a ratio of (3 pak choy : 1 sweet basil); (b) Photograph of pak choy plants showing increased biomass observed in pak choy upon exposure to 100 µM eugenol. Scale bar represents 5 cm. (c) Bar graph shows increase in average biomass as a result of the eugenol treatment. Individual data points are shown as dots and error bars represent standard deviations from n = 12 independent experiments.

**Supplementary figure 6** (a) Growth of 4-week-old pak choy plants under decreasing nitrate levels: 2.5 mM (full strength), 1.5 mM, 1 mM, 0.5mM and 0 mM and (b) Bar graph showing the biomass of Pak choy at different nitrate levels (n=4)

**Supplementary figure 7** Overview of MATLAB image processing, data visualization and temporal data extraction workflow for H_2_O_2_ and SA nanosensor infiltrated pak choy plants

**SUPPLEMENTARY TABLES**

**Supplementary Table 1** Modelled kinetic parameters and threshold concentrations ($K_{D}$) for the VOC-signaling model of pak choy intercropped with spearmint and sweet basil companion emitter plants.

**Supplementary Table 2** Major VOCs emitted by spearmint, sweet basil and pak choy plants using GC-MS SPME.

**Supplementary Table 3** RT-qPCR validation of selected DEGs from significant pathways

**Supplementary Table 4** Forward and reverse primer sequences of selected DEGs

**References**

(1) Stephenson, R. M. *Handbook of the thermodynamics of organic compounds*; Springer Science & Business Media, 2012.

(2) Stull, D. R. Vapor pressure of pure substances. Organic and inorganic compounds. *Industrial & Engineering Chemistry* **2002**, *39* (4), 517-550.

(3) Lugg, G. Diffusion coefficients of some organic and other vapors in air. *Analytical Chemistry* **1968**, *40* (7), 1072-1077.

(4) Anum, H.; Wang, Y.; Li, Y.; Sun, G.; Luo, J.; Gruda, N. S.; Liu, G.; Tong, Y. Physiological and nutritional responses of two pakchoi (Brassica chinensis) cultivars to different red-blue light ratios in controlled environment. *Frontiers in Sustainable Food Systems* **2025**, *9*, 1561118.

(5) Marino, S.; Ahmad, U.; Ferreira, M. I.; Alvino, A. Evaluation of the effect of irrigation on biometric growth, physiological response, and essential oil of Mentha spicata (L.). *Water* **2019**, *11* (11), 2264.

(6) Lim, S.; Kim, J. Light quality affects water use of sweet basil by changing its stomatal development. *Agronomy* **2021**, *11* (2), 303.

(7) Huang, H.; Hu, Y.; Wang, L.; Li, F.; Shan, Y.; Lian, Q.; Jiang, Y. Comparative profiles of the cuticular chemicals and transpiration barrier properties in various organs of Chinese flowering cabbage and Chinese kale. *Physiologia Plantarum* **2022**, *174* (2), e13650. DOI: <https://doi.org/10.1111/ppl.13650>.

(8) Schuster, A.-C.; Burghardt, M.; Riederer, M. The ecophysiology of leaf cuticular transpiration: are cuticular water permeabilities adapted to ecological conditions? *Journal of experimental botany* **2017**, *68* (19), 5271-5279.

(9) Wang, C. Y. Leafy, floral, and succulent vegetables. In *Postharvest physiology and pathology of vegetables*, CRC Press, 2002; pp 677-704.

(10) Munawar, A.; Zhang, Y.; Zhong, J.; Ge, Y.; Abou El-Ela, A. S.; Mao, Z.; Ntiri, E. S.; Mao, L. J.; Zhu, Z.; Zhou, W. Heat stress affects potato's volatile emissions that mediate agronomically important trophic interactions. *Plant, cell & environment* **2022**, *45* (10), 3036-3051. DOI: 10.1111/pce.14416 From NLM.

(11) Vettikkat, L.; Miettinen, P.; Buchholz, A.; Rantala, P.; Yu, H.; Schallhart, S.; Petäjä, T.; Seco, R.; Männistö, E.; Kulmala, M.; et al. High emission rates and strong temperature response make boreal wetlands a large source of isoprene and terpenes. *Atmospheric Chemistry and Physics* **2023**, *23*, 2683-2698. DOI: 10.5194/acp-23-2683-2023.

(12) Karban, R.; Maron, J.; Felton, G.; Ervin, G.; Eichenseer, H. Herbivore damage to sagebrush induces resistance in wild tobacco: Evidence for eavesdropping between plants. *Oikos* **2003**, *100*, 325-332. DOI: 10.1034/j.1600-0706.2003.12075.x.

(13) Karban, R.; Shiojiri, K.; Huntzinger, M.; McCall, A. C. Damage-induced resistance in sagebrush: volatiles are key to intra- and interplant communication. *Ecology* **2006**, *87* (4), 922-930. DOI: 10.1890/0012-9658(2006)87[922:drisva]2.0.co;2 From NLM.

(14) Mott, K. A.; Peak, D. Stomatal responses to humidity and temperature in darkness. *Plant, cell & environment* **2010**, *33* (7), 1084-1090. DOI: 10.1111/j.1365-3040.2010.02129.x From NLM.

(15) Hetherington, A. M.; Woodward, F. I. The role of stomata in sensing and driving environmental change. *Nature* **2003**, *424* (6951), 901-908. DOI: 10.1038/nature01843.
